# Spatial Proximity and Similarity of the Epigenetic State of Genome Domains

**DOI:** 10.1371/journal.pone.0033947

**Published:** 2012-04-04

**Authors:** Ekaterina E. Khrameeva, Andrey A. Mironov, Gennady G. Fedonin, Philipp Khaitovich, Mikhail S. Gelfand

**Affiliations:** 1 Institute for Information Transmission Problems, Russian Academy of Sciences, Moscow, Russia; 2 Faculty of Bioengineering and Bioinformatics, Moscow State University, Moscow, Russia; 3 Partner Institute for Computational Biology, Shanghai Institutes for Biological Sciences, Shanghai, China; 4 Max Planck Institute for Evolutionary Anthropology, Leipzig, Germany; National Institutes of Health, United States of America

## Abstract

Recent studies demonstrate that the organization of the chromatin within the nuclear space might play a crucial role in the regulation of gene expression. The ongoing progress in determination of the 3D structure of the nuclear chromatin allows one to study correlations between spatial proximity of genome domains and their epigenetic state. We combined the data on three-dimensional architecture of the whole human genome with results of high-throughput studies of the chromatin functional state and observed that fragments of different chromosomes that are spatially close tend to have similar patterns of histone modifications, methylation state, DNAse sensitivity, expression level, and chromatin states in general. Moreover, clustering of genome regions by spatial proximity produced compact clusters characterized by the high level of histone modifications and DNAse sensitivity and low methylation level, and loose clusters with the opposite characteristics. We also associated the spatial proximity data with previously detected chimeric transcripts and the results of RNA-seq experiments and observed that the frequency of formation of chimeric transcripts from fragments of two different chromosomes is higher among spatially proximal genome domains. A fair fraction of these chimeric transcripts seems to arise post-transcriptionally via trans-splicing.

## Introduction

DNA molecules are tightly packed within the mammalian nucleus, yet little is known about the chromatin organization beyond the scale of nucleosomes. We are just beginning to comprehend the complexity of the chromosome folding principles and how they might shape the transcriptional regulation. In interphase nuclei, chromosomes are organized into distinct, dynamic, non-overlapping territories [1]. The dynamic rearrangements of chromosome regions relative to other chromosomal loci appear to be involved in the regulation of gene expression. Advancing technological developments have revealed that the chromatin is folded into loops bringing together loci from different chromosomes. This observation has led to the hypothesis that genes can be regulated in trans by regulatory elements on other chromosomes [2].

The development of the chromosome conformation capture (3C) technology has enabled detailed analysis of long-range interactions in the chromatin. This method uses spatially constrained ligation followed by locus-specific polymerase chain reaction [3]. Recently, a new technology called Hi-C was developed. It probes the three-dimensional architecture of whole genomes by coupling proximity-based ligation with parallel sequencing [4]. The authors constructed a spatial proximity map of the human genome at the resolution of 1 megabase for the lymphoblastoid cell line GM06990 and the erythroleukemia cell line K562.

Another area where technological advances have generated huge amounts of data is the characterization of the functional state of the chromatin as reflected in the epigenetic marks such as methylation of DNA, histone modifications, and DNAse sensitivity demonstrating the open state of the chromatin [5]–[7]. It has been shown that these characteristics correlate with each other, as well as with localization of genes, promoters, enhancers and other functional regions, and gene expression levels [8]–[11]. A functional annotation of the human genome revealing the genome-wide locations of diverse classes of epigenetic mark combinations, or chromatin states, has been provided [12].

Here, we study correlations between spatial proximity of genome domains, located on different chromosomes, and their epigenetic marks. The results show that interacting loci seem to share transcriptional factories and have similar histone modifications, methylation state, DNAse sensitivity level, expression level, and chromatin state patterns in general.

Deep sequencing of transcriptomes from worms to humans reveals that some transcripts are composed of sequence segments that are not co-linear, with pieces of sequence coming from distant regions of the reference genome, even from different chromosomes [13]. Some of these chimeric transcripts are formed by genetic rearrangements, but others may arise post-transcriptionally via trans-splicing [14]. Recent studies suggest that apparent chimeric RNAs might possibly be generated by experimental artifacts [15]. However, the same study identified 80 genes undergoing trans-splicing between homologous alleles. Here, we observe numerous chimeric RNAs between spatially proximal regions of different chromosomes, suggesting that trans-splicing is a more common process in human than previously believed and may govern expression of architecturally complex genes.

## Results

### Elimination of systematic biases affecting Hi-C procedure

The spatial proximity map was constructed using high-throughput sequencing methods [4] and could be contaminated by sequencing artifacts originating from template switching during RT-PCR reaction or read mapping errors. Both these types of sequencing artifacts seem to occur more frequently in highly homologous regions. If there are two such regions located in two distant parts of the genome, mapping programs can get confused and align the second mate pair read to a different location instead of the locus the first mate pair read is aligned on. DNA-polymerase also can switch between template molecules if the latter contain stretches of identical sequences [16].

**Figure 1:**
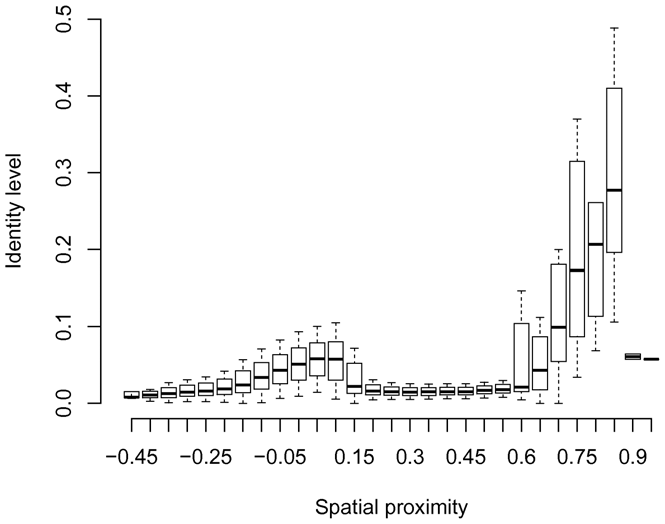
Sequence identity levels in 29 considered intervals of spatial proximity values between 1-Mb fragments of different chromosomes in the genome-wide correlation matrix 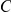
 [**4**] **(see** **Methods** **for the details).** Negative spatial proximity values correspond to fragments distant from each other, positive values correspond to proximal fragments. The whisker boxes show quartiles, median (the line in the box), min and max values (the lines outside the box).

**Figure 2:**
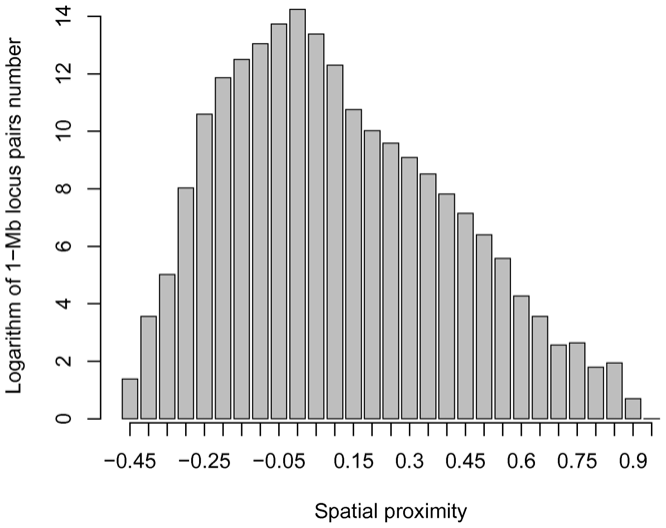
Histogram of the number of pairs of interacting genome fragments originating at different chromosomes in the human genome-wide spatial proximity matrix 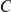
 **.**

To control for these possibilities, we calculated sequence identity levels between interacting genome fragments (Fig. 1). Only pairs of fragments originating at different chromosomes were considered here and in all further analyses to avoid normalization for the linear distance between the fragments. The identity level for two interacting fragments 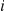 and 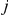 was computed by summing the length of sequence regions highly similar between these fragments (found by the *blastn* tool [17] with 92% threshold for identity that is equivalent to a 75 nucleotide read with 6 mismatches) and dividing it by 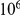. Spatial proximity values were divided into 29 intervals. Abnormally high identity levels were observed in genome fragments with the spatial proximity values higher than 0.55, leading to the conclusion that some of these “spatially proximal” fragments may not be adjacent in the nucleus but result from sequencing or mapping artifacts, such as PCR recombination events in the Hi-C protocol. Moreover, the total number of fragment pairs in the tail intervals is much lower than in the central intervals (Fig. 2), and the results for these intervals have lower statistical robustness. Hence, only intervals from 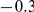 to 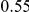 are considered further.

We also observed a distinct, although non-significant, peak of the similarity between fragments with spatial proximity near zero. To identify its source, we analyzed the repeat content of fragments using the data from the UCSC Genome Browser Database [18] (Fig. S1). The repeat content was calculated as the average number of nucleotides masked by *RepeatMasker* program in two interacting 1-Mb fragments. A peak in the near zero interval was observed for exapted repeats (conserved non-exonic elements that have been deposited by mobile elements [19]). That might mean that such repeats are slightly underrepresented in “non-standard” regions distant from the rest of the chromatin, or forming tight foci. This observation deserves a special, separate analysis.

After this manusript had been submitted, another paper describing biases in the Hi-C data was published [20]. The authors report on the distance between restriction sites, the GC content of the ligation junctions and read mappability as the major systematic biases affecting the Hi-C experimental procedure. To eliminate these biases, they developed an algorithm re-normalizing the Hi-C data. Following this algorithm, we repeated their normalization procedure and produced the genome-wide smooth normalized contact enrichment matrix 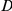 for the lymphoblastoid cell line GM06990, exactly as described in [20]. We repeated our analyses (see below) for this matrix 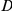 and two matrices from the original Hi-C paper [4] (the contact enrichment matrix 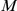
* and the correlation matrix 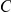, see Methods for details) independently to demonstrate that the results do not depend in principle on a smoothing algorithm, normalization method, or bias removal procedure.

### Similarity of epigenetic marks in spatially proximal domains

We tested whether frequently interacting fragments have a similar level of histone modifications, methylation state, DNAse sensitivity, and expression using the data from several high-throughput studies (see “Methods”). All these features have a similar data structure that is represented by markers along the genome. Being measured, each marker is characterized by a peak of a defined width and height (signal). To measure signal strength 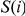 over each 1-Mb fragment 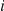, we multiplied the height of each peak 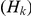 by the fraction of the fragment 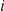 intersecting with the peak 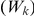, and then summed up the results for all peaks 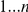 in the locus 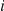:
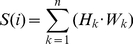
(1)Though some epigenetic marks are punctual (such as H3K4me3 at the promoter region) while others denote domains (such as H3K36me3 throughout the gene body), their fine structure is unlikely to affect the signal strength 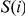 value at the coarse 1-Mb resolution. The difference 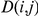 of signal strength between two interacting fragments 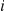 and 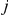 was calculated as:
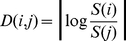
(2)This definition does not depend on the order of the considered fragments; the signal values are non-negative, and hence the logarithm is well-defined.

**Figure 3:**
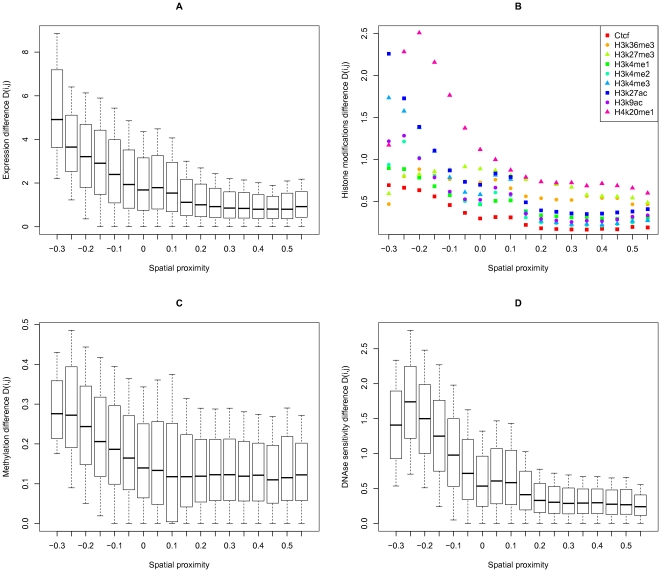
Correlations of the spatial proximity values with expression (A), histone modifications (B), DNA methylation (C), and DNAse sensitivity (D) differences. The whisker boxes (A,C,D) are as in Fig. 1. Symbols in B show the medians for different histone modifications; the whisker boxes for all modifications are given in Fig. S1.

The median 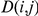 value was calculated for each considered interval of spatial proximity in the correlation matrix 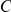. These values correlate with the spatial proximity values for each data type we tested: expression level (Fig. 3A, Spearman's rho = 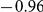, p-value = 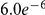), histone modifications (Fig. 3B,Fig. S2, average Spearman's rho = 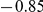, average p-value = 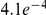), DNA methylation (Fig. 3C, Spearman's rho = 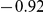, p-value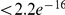), DNAse sensitivity (Fig. 3D, Spearman's rho = 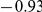, p-value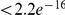). UCSC snapshots of examples for the each evaluated feature are shown in Fig. S3.

The same procedure was repeated for the contact enrichment matrices 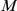
* and 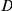. Spatial proximity values were divided into 29 intervals (Fig. S4). As for the contact matrix 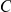, 18 central intervals were selected for further analysis. Both matrices 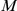
* and 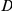 demonstrate strong correlations with the spatial proximity values for each data type we tested: expression level (Fig. S5A and S6A, Spearman's rho = 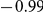 and 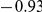, p-value = 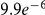 and 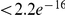 for 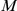
* and 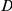, respectively), histone modifications (Fig. S5B and S6B, average Spearman's rho = 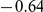 and 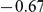, average p-value = 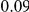 and 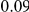), DNA methylation (Fig. S5C and S6C, Spearman's rho = 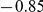 and 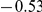, p-value = 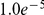 and 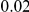), DNAse sensitivity (Fig. S5D and S6D, Spearman's rho = 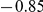 and 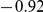, p-value 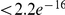 and 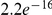). As all three matrices 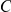, 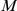
* and 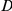 show approximately the same results, we selected only one of them, matrix 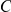, for further analysis.

### Functional similarity of genes in spatially proximal domains

To check if the observed correlations can be extended to the Gene Ontology level, we studied semantic similarity of GO terms between interacting genome fragments. Each 1-Mb fragment was assigned a list of GO terms corresponding to genes of this fragment. To calculate the average GO semantic similarity between 1-Mb fragments 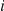 and 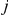, we composed an 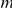-by-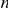 matrix 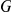 for each pair of fragments 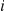 and 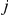, where 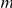 is the length of GO term list for the fragment 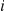, and 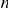 is the length of GO term list for the fragment 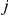. Elements of the matrix 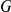 were calculated with the package GOSemSim [21], which computes the semantic similarity between two GO terms using Wang's graph-based algorithm [22]. Then the average value of the matrix 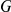 was calculated.

**Figure 4:**
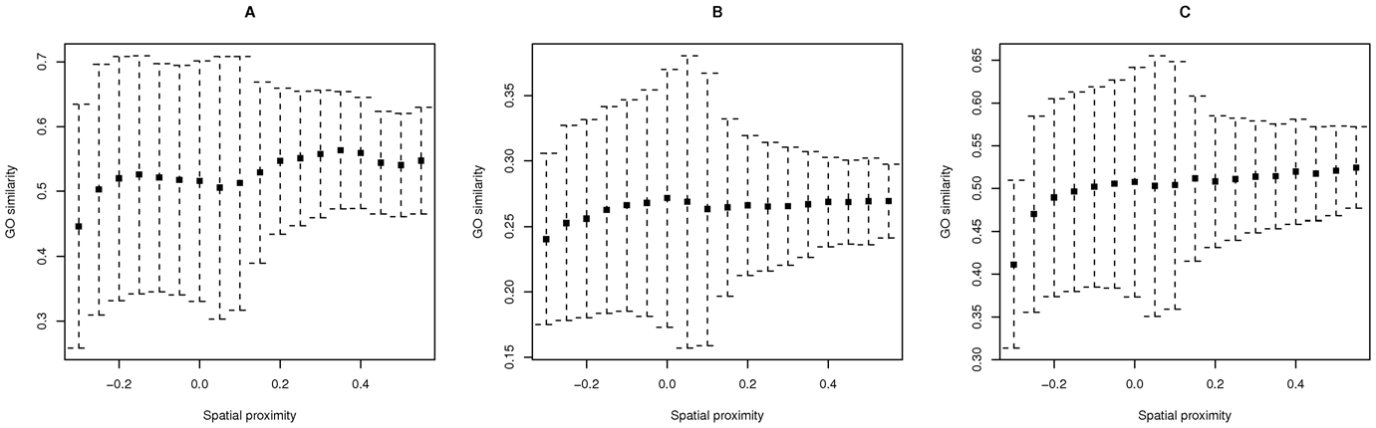
Correlation of the spatial proximity values with the Gene Ontology semantic similarity of the genes located in the interacting genome fragments. (A) Molecular Function. (B) Biological Process. (C) Cellular Component. Black squares show average GO similarity values, dashed lines, standard deviations.

The procedure described above was repeated for the Molecular Function, Biological Process and Cellular Component hierarchies separately. The average GO semantic similarity appeared to correlate with the spatial proximity values for all GO hierarchies (Fig. 4A–C, Spearman's rho = 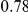, 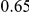, 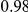, p-value = 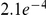, 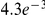, 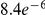, respectively), with the highest correlation coefficient for the Cellular Component hierarchy.

### Co-expression of genes in spatially close fragments

We also studied co-expression of spatially close fragments. The COXPRESdb database [23] was used as a source of the co-expression data. To measure average co-expression 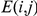 for two interacting 1-Mb genome regions 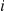 and 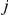, we used formula [3] for all gene pairs 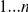 linked in COXPRESdb:
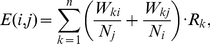
(3)where 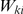 is the fraction of the 1-Mb locus 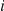 intersecting with the first gene of the linked pair 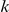, 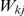 is the fraction of the 1-Mb locus 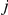 intersecting with the second gene of the pair k, 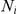 is the number of genes in the locus 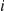, 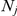 is the number of genes in the locus 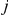, 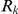 is Pearson's correlation coefficient between expression profiles of the linked gene pair 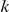.

**Figure 5:**
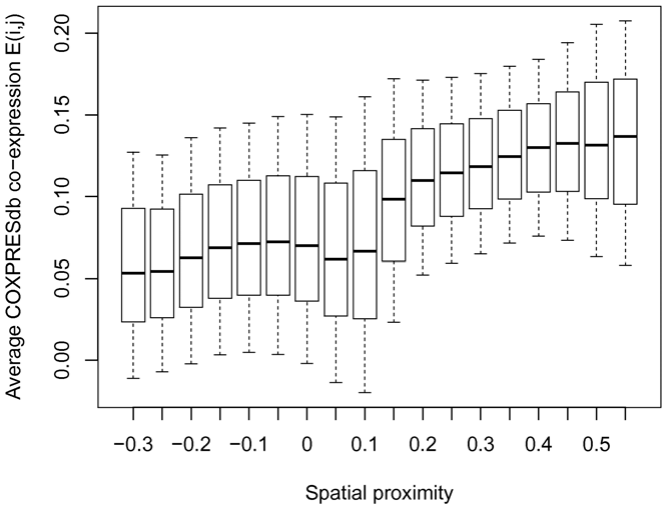
Correlation of the spatial proximity values with COXPRESdb co-expression values. **Whisker boxes are as in** **Fig. 1** **.**

The median 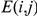 value was calculated for each considered interval of spatial proximity. A strong correlation with the spatial proximity values was observed (Fig. 5, Spearman's rho = 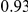, p-value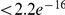).

### Consistency with the two-compartment chromatin model

**Figure 6:**
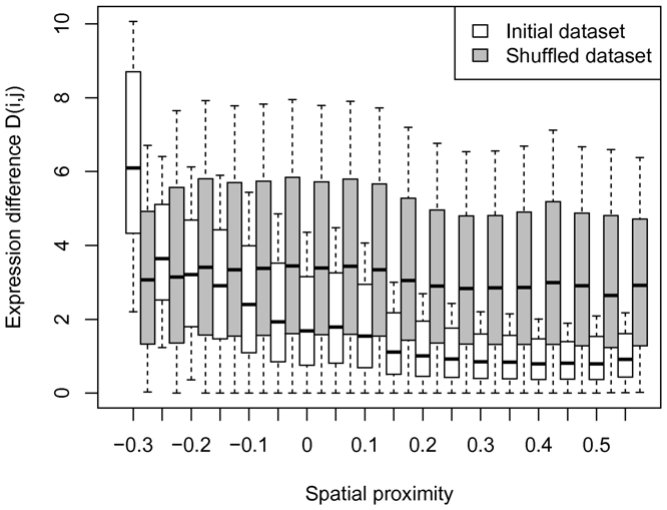
Correlations of the spatial proximity values with the expression level difference in the initial dataset and in the shuffled dataset.

According to the two-compartment chromatin model provided by [4], the entire genome can be partitioned into two spatial compartments: compartment A associated with open, accessible, actively transcribed chromatin, and compartment B with the opposite characteristics. To test for the consistency with this model, we produced a control dataset in which we shuffled only gene names within the two compartments independently while retaining gene positions to keep gene-rich and gene-poor genome domains intact (see “Methods”). Such type of the control dataset was applied because there is a correlation between the gene content and the spatial proximity of genome domains (Fig. S7, Spearman's rho = 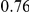, p-value = 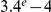). Hence one could suppose that the observed similarity of epigenetic marks in spatially proximal domains might be caused by their location in the gene-rich open chromatin compartment. Our control dataset keeps gene content and compartments intact, yet correlations drop considerably (average Spearman's rho = 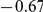, average p-value = 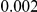, compare to 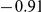 and 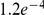, respectively, for the initial dataset), meaning that the observed epigenetic similarity of spatially proximal fragments cannot be simply explained by either their common origin from the same chromatin compartment or the higher gene content. Moreover, in the shuffled dataset the entire profile of the dependency between the spatial proximity values and the evaluated epigenetic features looks completely different. The 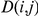 difference values increase several-fold for spatially proximal fragments (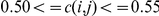) and decrease for spatially distant ones (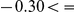
c(i,j)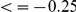). See an example for the expression level difference in Fig. 6).

Additionally, we divided pairs of interacting 1-Mb fragments from different chromosomes into three groups: (1) both fragments are in the closed-chromatin compartment; (2) both fragments are in the open-chromatin compartment; (3) the fragments are in the different compartments. The main calculations were repeated for these groups independently and it appeared that epigenetic similarity of spatially proximal fragments is observed in each of the three groups and does not depend on the compartment of origin (Fig. S8).

### Spatially proximal domains share chromatin state patterns

**Figure 7:**
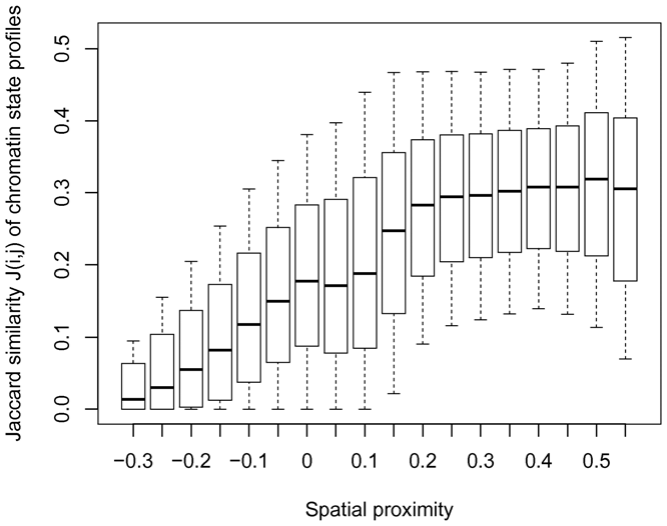
Correlation of the spatial proximity values with the similarity of chromatin state profiles. **Whisker boxes are as in** **Fig. 1** **.**

We then considered ‘chromatin states’, biologically-meaningful combinations of chromatin marks [12]. To compare chromatin state profiles between two interacting 1-Mb fragments, we assigned a vector of length 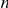, where 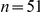 is the number of chromatin states, to each 1-Mb fragment. Element 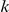 was defined as the fraction of the 1-Mb fragment annotated as 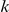-th chromatin state, 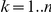. Similarity of two such vectors 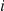 and 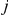 was calculated using the Jaccard similarity coefficient as:
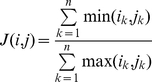
(4)
Fig. 7 shows that the spatial proximity values are strongly correlated with the Jaccard similarity of chromatin state profiles (Spearman's rho = 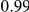, p-value = 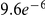).

### Spatial clustering yields functionally homogeneous domains

**Figure 8:**
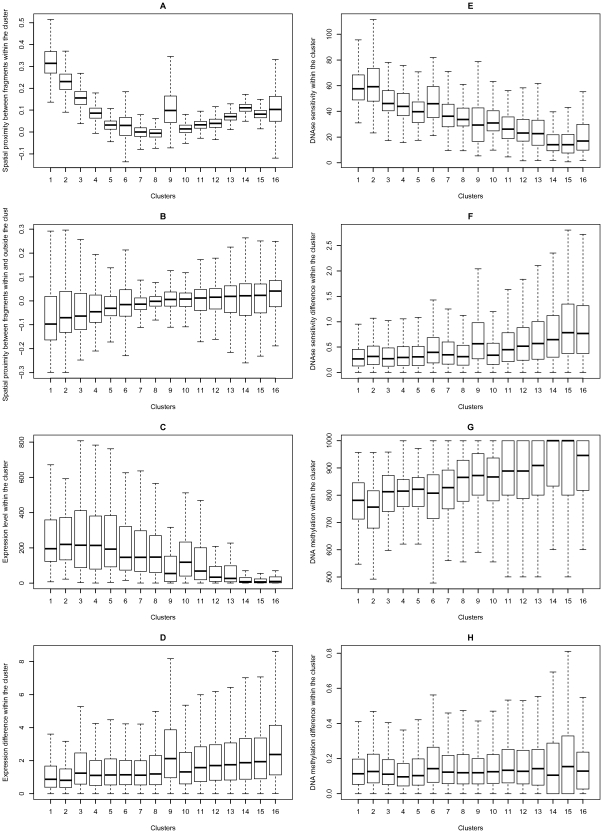
DNA fragments clustered into 16 groups. (A) spatial proximity between all possible pairs of DNA fragments within the cluster; (B) spatial proximity between each DNA fragment within the cluster and each DNA fragment from the remaining set; (C,E,G) expression, DNAse sensitivity, and DNA methylation levels within the cluster; (D,F,H) expression, DNAse sensitivity, and DNA methylation differences within the cluster. See Fig. S10, S11, S12, S13 for DNA fragments clustered into 4, 8, 32, and 64 groups, respectively.

DNA fragments in the spatial proximity map were clustered into several groups (4, 8, 16, 32, and 64 groups; see Table S2) by Ward's minimum variance method [24] in the R environment [25]. For each cluster, we calculated spatial proximity between all possible pairs of DNA fragments within the cluster (Fig. 8A for DNA fragments clustered into 16 groups); spatial proximity between each DNA fragment within the cluster and each DNA fragment outside the cluster (Fig. 8B); expression level, histone modifications, DNA methylation, DNAse sensitivity, and their differences within the cluster (Fig. 8C–H,Fig. S9). See Fig. S10, S11, S12, S13 for DNA fragments clustered into 4, 8, 32, and 64 groups, respectively.

The results show that the median spatial proximity between DNA fragments within the cluster is anti-correlated with the median spatial proximity between DNA fragments within and outside the cluster (Pearson's correlation coefficient = 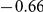, p-value = 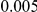). One may conclude from Fig. 8A and B that some clusters (‘compact clusters’) have high spatial proximity values within a cluster, being located at a distance from the remaining clusters in the nucleus, while other clusters (‘loose clusters’) have low spatial proximity values within a cluster, being located at more or less average distance from the remaining clusters. Moreover, the compact clusters are actively transcribed because expression, histone modifications, and DNAse sensitivity levels are higher in these clusters, while the methylation level is lower. On the contrary, average differences in expression, histone modifications, and DNAse sensitivity are lower in compact clusters, meaning that the genome fragments in these clusters have not only high but also similar levels of expression, histone modifications and DNAse sensitivity.

Fig. S14 demonstrates that compact clusters have slightly higher linear proximity within a cluster than other clusters as the spatial proximity is weakly correlated with the linear proximity within a cluster (Pearson's correlation coefficient = 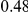, p-value = 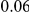). This observation can be explained by a slightly higher number of the genome fragments originating at the same chromosome in compact clusters.

### Functional similarity implies spatial proximity

To characterize further the relationship of the chromatin properties with the spatial proximity, we built linear regression models, which predict spatial proximity of a pair of fragments using the differences and averages of signal strength in these fragments, the signal set comprising all 12 studied chromatin properties. Accuracy of each model was estimated by two-fold cross-validation and compared with the accuracy of a naive algorithm that produces the average spatial proximity on the training set as the predicted value. The difference of the signal strengths was computed using (2), the average was computed as:
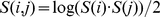
(5)


Calculations showed that the use of the 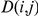 values (differences) results in significant accuracy gains over the naive algorithm for all properties (Table S1). The use of 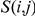 values (averages) also yielded significant accuracy gain for all properties except histone modifications H3K27me3, H3K4me2, H3K4me3. For H4K20me1, expression, DNAse sensitivity and methylation state the accuracy gain is higher with averages than with differences. The spatial proximity is anti-correlated with all differences and is positively correlated with all averages except methylation, meaning that the spatially proximal genome fragments are actively transcribed and have similar epigenetic marks, while spatially distant fragments have opposite characteristics.

**Figure 9:**
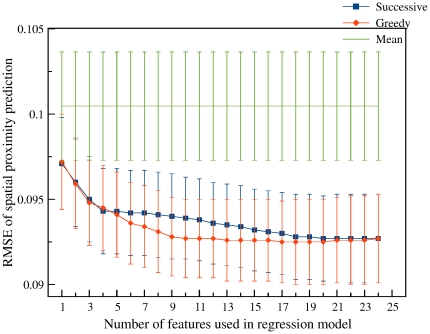
The root mean squared error (RMSE) of the spatial proximity prediction with standard deviations (SD) (all values were estimated on the testing set for each split and averaged) of regression models, which used one through 24 features, selected by two algorithms: Successive (successive selection based on individual accuracy) and Greedy (greedy forward feature selection), and the RMSE with SD of an algorithm, which always uses the training set mean as the predicted value. Additional information about used features can be found in Table S1.

Further, regression models, which simultaneously use one through 24 difference and average values of all properties as features, were built. Features were added successively either in order of decreasing accuracies of single-feature models, or by the greedy algorithm, which adds the features that yield the highest accuracy gain on every iteration. The root mean squared error (RMSE) values of models on the testing set for various feature sets are presented in Fig. 9. The maximal decrease of the prediction error during successive feature selection was obtained when the first four stably selected (i.e. added at the first four steps for all considered sample splits, see Methods) features were used. These features are the averages and differences of DNAse sensitivity and H4K20me1 modification. Further addition of features leads to slow decrease of the prediction error until the addition of the 18th feature. After that the prediction quality does not change. However, for different sample splits, different features are added. The greedy feature selection reaches the plateau faster: the error stops decreasing after addition of the 8th feature. Most frequently selected features are the differences and averages of DNAse sensitivity, histone modification H4K20me1, CTCF density, methylation state. However, only the first three features are stably selected. These features are the same as leaders of successive selection, excluding the H4K20me1 averages.

To control for the consistency with the two-compartment chromatin model provided by [4], we divided pairs of interacting 1-Mb fragments from different chromosomes into three groups: (1) both fragments are in the closed-chromatin compartment; (2) both fragments are in the open-chromatin compartment; (3) the fragments are in the different compartments. The regression models were built for these groups independently, and similar results were observed in each of the three groups (Fig. S15, S6, S17). Hence, one can conclude that the similarity of functional states may be used to predict the spatial proximity of fragment pairs, independently of the compartment of origin.

To visualize the relation between the spatial proximity and features and the regression models, plots of aggregated sample values were built (Fig. S18, S19, S20, S21, S22, S23, S24, S25). The aggregation was performed by ordering all pairs of fragments by the increase of the value of the considered feature, grouping all pairs successively according to this order (a group size was set to 50000 pairs) and then averaging feature and proximity values of all members of each group (which results in 15 points for each feature). The model parameters were estimated on the full sample and corresponding lines were added on the plots.

### Spatially close fragments produce chimeric RNAs

To retrieve candidate chimeric RNAs, paired reads of three transcriptomic RNA-Seq datasets (brain tissue [26], lymphoblastoid cell line GM12878 [27], and erythroleukemia cell line K562 [28]) were mapped to the human reference genome and to all possible intragenic splice junctions (see “Methods”). Chimeric pairs consisting of reads that map to different chromosomes were selected for further analysis. There were 431321 such pairs for the brain tissue sample, 907368 pairs for the GM12878 cells, and 361487 pairs for the K562 cells.

**Figure 10:**
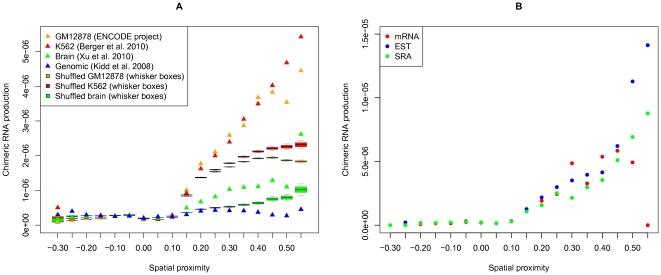
Correlation between chimeric RNA production and spatial proximity values for (A) the K562 cell line, the GM12878 cell line, and the brain tissue sample (red, orange and green triangles, respectively); the genomic rearrangement dataset (shown in blue); the shuffled control K562 dataset (red whisker boxes); the shuffled control GM12878 dataset (orange whisker boxes); the shuffled control brain dataset (green whisker boxes) and (B) three ChimerDB datasets: mRNA, EST and SRA-derived (red, blue and green dots, respectively).

We tested the brain tissue, GM12878 and K562 data against the chromatin spatial proximity matrix. Eighteen intervals of the spatial proximity values were considered. For each interval, we calculated the fraction of fragment pairs in which we observed chimeric read pairs. To make different datasets comparable, this value was further divided by the total number of chimeric pairs in the sample. All three datasets show significant correlations between the frequency of chimeric RNAs and the spatial proximity (Spearman's rho = 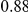, 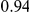, 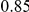, p-value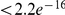, 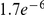, 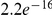, respectively), in comparison to the corresponding control datasets (Fig. 10A). The control datasets were produced by re-pairing of each read with a random read on a different chromosome (see Methods for details). The observed weak correlation between the chimeric RNA production in the brain tissue and the spatial proximity values in the lymphoblastoid cell line is quite remarkable because this could mean that the three-dimensional architecture of chromosomal interactions is, at least partially, similar in such diverse cells as lymphoblasts (even represented by different cell lines) and neurons.

The observed correlations in both datasets could be caused by (at least) two reasons: trans-splicing and genome rearrangement. To test these possibilities we analyzed the data on genome trans-chromosomal rearrangements from [29] and observed no increase in the number of chimeric pairs among spatially close regions (Fig. 10A). Hence, there remains a distinct possibility that the observed chimeric transcripts indeed originate from trans-splicing.

In the same way, we also associated the spatial proximity values with the ChimerDB database [30] that contains chimeric transcripts collected from various public resources. Fig. 10B shows that the spatial proximity values correlate with the production of chimeric transcripts derived from all three data sources (EST, SRA, mRNA) available in ChimerDB (Spearman's rho = 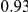, 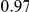, 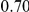, p-value 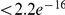, 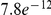, 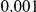, respectively).

## Discussion

The observed correlations between the spatial proximity of genome regions and a variety of their functional characteristics seem to demonstrate the presence of co-regulated genome domains formed by regions of different chromosomes. All correlations are significantly higher than in corresponding control datasets.

Our choice of the control procedure stemmed from the possibility that the observed correlations of the expression level and other characteristics with the spatial proximity could be explained by the fact that there are two types of chromatin foci, loose gene-poor and dense gene-rich ones. One could suppose that gene-poor 1-Mb genomic fragments are located far from other chromosomal regions and their median 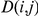 values would consequently strongly differ from the median 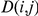 values of gene-rich 1-Mb fragments. Indeed, we observed a correlation between the gene content and the spatial proximity of genome domains. This correlation could further lead to a correlation between spatial proximity and median histone modifications, methylation state, DNAse hypersensitivity, and expression 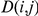 values.

However, the control procedure, implemented here, shuffles only gene names and does not affect the gene content. Yet we observe significantly higher correlations in the original, non-shuffled datasets than in the control. It means that the non-uniformity of the gene content only partially explains the observed correlations. It seems that genome domains with similar functional patterns, located on different chromosomes, tend to be spatially close to each other so that they can share transcription factories. This association does not depend solely on the chromosomal territories or the local gene content.

Correlation between the gene content and the spatial proximity agrees with the existing understanding of the chromatin organization in the interphase nucleus. It is widely accepted that active gene-rich chromosome regions assume more interior positions in the nucleus, whereas the nuclear periphery generally harbors mainly gene-poor chromosome regions [31]. Hence, one could assume that transcriptionally active gene-rich regions would tend to locate near other gene-rich regions, even if the latter are located on a different chromosome, sharing their transcription machinery, factors and regulatory elements. Therefore, these interacting loci could easily have similar chromatin state.

However, our observations cannot be reduced to this simple explanation. In the control dataset, gene names were shuffled within the open/closed chromatin compartments independently, according to the annotation provided by [4]. If the open/closed chromatin model was sufficient, one would expect to find high correlations with the epigenetic features in the control dataset. Yet weak correlations were observed. Moreover, spatially proximal fragments have more similar epigenetic state than distant ones, independent on the compartment of origin (both fragments are in the closed-chromatin compartment, both fragments are in the open-chromatin compartment, or both fragments are in the different compartments). It proves that the observed presence of co-regulated genome domains formed by regions of different chromosomes cannot be simply explained by their common origin from the open-chromatin compartment. Interestingly, the first group (both fragments are in the closed-chromatin compartment) demonstrates low similarity of the epigenetic state at high spatial proximity values, which is expected as closed chromatin regions are transcriptionally inactive and unlikely to share transcription factors, regulatory or other elements of the transcription machinery.

Additionally, when the chromosomal regions were clustered by spatial proximity, two general types of functional regions emerged. Compact clusters with high spatial proximity between fragments within a cluster and relatively low spatial proximity with regions belonging to other clusters were characterized by higher expression rates, histone modification and DNAse sensitivity levels, while the level of methylation in such clusters was lower. Loose clusters with low spatial proximity between fragments had opposite characteristics. Compact clusters likely correspond to the foci of active transcription (transcription factories). At that, it should be recalled that in all our analyses we considered only pairs whose constituents originated at different chromosomes, and thus these results do not depend on the local, linear proximity along a chromosome. As an additional check, we calculated the linear proximity within the clusters and it appeared to correlate with the spatial proximity only weakly (not significant at the 5% confidence level).

The linear regression analysis revealed that the similarity of functional states may be used to predict the spatial proximity of fragment pairs. At that, both averages and differences of the parameters of paired fragments are important, measuring the overall state and the differences that reflect functional homogeneity. The most informative features for such analysis are the DNAse sensitivity and, surprisingly, the histone modification H4K20me1, followed by the CTCF density and the methylation state. While the DNAse sensitivity, the CTCF density, and the methylation state are known to be associated with the chromatin structure and transcription activity [8], [9], the function of the H4K20me1 mark is not yet well-established.

There is evidence that H4K20me1 can be important for programmed genomic rearrangements [32]. Also, the N tail of histone H4 is essential for the chromatin structure packing [33], and only lysine 20 can be methylated in mammalian cells. The relationship between the H4K20me1 mark and the transcription activity remains controversial [34]. There are studies that link H4K20me1 with the transcription level [35], [36] and several papers demonstrate strong dependency between the H4K20me1 mark and the transcriptional repression [37], [38]. Most likely, H4K20me1 is a very dynamic histone modification and can play different roles at the different cell-cycle stages. The strong correlation between the H4K20me1 mark and the spatial proximity observed here is yet another evidence of H4K20me1 involvement in both chromatin structure and gene expression regulation.

The chimeric RNAs are a popular subject, as most of them are known to be produced by cancer cells [39]. However, evidence of chimeric RNAs in normal cells is also starting to emerge [40]. The origin of the chimeric RNA molecules is not clear yet. There are at least three possibilities: genomic rearrangements relative to the reference genome, trans-splicing, and cloning or sequencing artifacts. It seems that all these sources contribute to the accumulated chimeric RNA data [14]. Here we demonstrate a possibility of trans-splicing. Indeed, the analysis of the genomic data shows that genomic rearrangements cannot explain the observed frequency of chimeric RNAs.

Further, the generation of chimeric RNAs in eukaryotes is known to be strongly associated with short homologous sequences at chimeric RNA junction sites [41]. Hence, the control for sequencing artifacts, implemented here for the spatial proximity map validation, is also relevant to the chimeric RNAs. As identity levels are not elevated in all considered intervals of spatial proximity, we believe that sequencing artifacts do not influence chimeric RNA production significantly. Hence, the observed chimeric transcripts may originate, at least partially, from trans-splicing between different chromosomes.

The observed correlations between the spatial proximity values and the production of chimeric transcripts retrieved from ChimerDB are quite remarkable. ChimerDB is the most complete and up-to-date knowledgebase of fusion transcripts collected from a variety of tissues. The strong correlations with the spatial proximity values for the lymphoblastoid cell line most likely mean that the three-dimensional architecture of chromosomal interactions is sufficiently similar for different cell types to retain the observed correlation. Indeed, interchromosomal spatial proximity values for the lymphoblastoid cell line GM06990 and erythroleukemia cell line K562 are strongly correlated (Pearson's correlation coefficient = 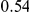, p-value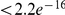). Moreover, we observed a strong correlation between the spatial proximity values and the chromatin state profiles though the chromatin states were annotated for CD4 T-cell line.

This allows us to conclude that the 3D structure of the nuclear chromatin seems to demonstrate consistent patterns throughout the human body and contains co-regulated genome domains formed by regions of different chromosomes that share various epigenetic features, have equally high expression level and can produce fusion transcripts.

## Methods

### Chromatin functional states

Results of several high-throughput studies were used to retrieve the chromatin functional state data. Expression data for the lymphoblastoid cell line GM06690 were obtained from [4]. Histone modifications, methylation state, and DNAse sensitivity data were obtained from the ENCODE project for the lymphoblastoid cell line GM12878 [27]. Spatial proximity values were extracted from the genome-wide spatial proximity map for the lymphoblastoid cell line GM06990 [4].

Genome-wide annotation of ‘chromatin states’, or biologically-meaningful combinations of chromatin marks, was derived from [12]. The annotation included 51 chromatin states and was based on a set of 38 different histone methylation and acetylation marks in human CD4 T-cells, as well as histone variant H2AZ, PolII, and CTCF5.

Two datasets of human transcriptomic samples (brain tissue [26] and erythroleukemia cell line K562 [28]), as well as the ChimerDB database [30] were used to retrieve candidate chimeric RNAs. To do that, paired reads were mapped to the human reference genome (version hg18) and to all possible intragenic splice junctions with the SOAP program [42].

### 3D chromosomal interactions

In the spatial proximity map 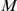 of the human genome, constructed by the Hi-C method [4], an entry 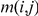 is defined to be the number of ligation products between fragments 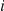 and 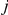. In [4], the matrix 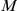 was normalized for coverage and a new matrix 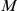
* was produced. Only fragment pairs originating at different chromosomes were considered. The expected number of interactions between each fragment pair 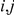 was computed by multiplying the fraction of reads containing 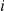 by the fraction of reads containing 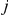 and multiplying by the total number of reads. The enrichment was computed by taking the actual number of interactions observed between fragment 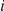 and fragment 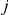, 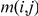, and dividing it by this expected value. To improve the resolution, the correlation matrix 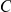, in which 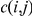 is Pearson's correlation coefficient between the 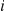-th row and the 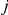-th column of 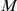
*, has been constructed.

### Control procedures

To control for the influence of gene-rich and gene-poor genome domains, we shuffled gene names, while retaining gene positions. This procedure rearranges signal values only and does not affect correlations between the spatial proximity and the gene content in pairs of genome regions. According to the open/closed chromatin annotation provided by [4], we assigned each gene to a chromatin compartment containing the start of the coding region of this gene, and shuffled gene names only within the same compartment.

For the chimeric RNA dataset, the following control procedure was applied. Each read pair, consisting of reads mapping to the same chromosome, was unpaired. The unpaired reads were randomly paired with unpaired reads on a different chromosome. The resulting dataset, consisting of artificial chimeric RNAs, also retains the original gene content, as the local read coverage is not changed.

### Regression models and feature selection

Ridge regression models [43] were used as the regression model. To estimate the models' accuracy, the list of studied fragments was randomly split in two equal parts, one used to build the training set, and the other, the testing set. Then these parts were exchanged. The splitting was repeated 100 times. At each split, fragment pairs with both members belonging to the training or testing list, were used as the training and testing sets, respectively. The root mean squared deviation of the predicted value from the real spatial proximity (RMSE) was computed on the testing set. Then, all error values obtained for different sample splits were averaged. The splits were fixed during testing of all algorithms. To compare the regression accuracy with the accuracy of prediction based on the average value, p-values of paired, two-sample, two-tailed T-test with the Bonferroni correction for multiple comparisons were used. P-values 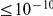 were considered significant. For successive feature selection, the training set was randomly split in two equal parts. The regression model was trained on one part for each feature, and the model accuracy was estimated on the other part. The splitting was repeated 10 times. After that, all features were ordered by the increase of average RMSE. During the greedy selection, sample splits and accuracy estimation were done in the same way. At the 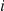-th iteration, all features, not belonging to the list of already selected features of length 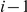, were added to this list one at a time, the regression models were trained using the obtained feature lists of length 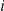, and the prediction error was computed on the testing set. The list yielding the model with the smallest average error was selected as the current list of the selected features of length 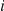. The selection stability was estimated by frequencies of 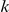 most frequently selected features in the lists of length 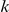 (for all 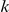). A feature was considered to be stable if its frequency was close to one.

## Supporting Information

Figure S1 **The average repeat content in 29 considered intervals of the spatial proximity in the the genome-wide correlation matrix C.** (A) Simple repeats. (B) Nested repeats. (C) Exapted repeats.
(TIFF)

Figure S2 **Correlations of the spatial proximity values with histone modifications differences. All notations are as in** **Fig. 3** **.**
(TIFF)

Figure S3 Examples of spatially proximal (chr3:49000000–49999999 and chr11:66000000–66999999, spatial proximity = 0.54) and spatially distant (chr3:49000000–49999999 and chr4:164000000–164999999, spatial proximity = −0.25; chr4:164000000–164999999 and chr11:66000000–66999999, spatial proximity = −0.22) fragments. Spatially proximal fragments have similar epigenetic features, while spatially distant have rather different ones.
(TIFF)

Figure S4
**Histograms of the number of pairs of the interacting genome fragments originating at different chromosomes in the human genome-wide spatial proximity matrices** 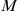 * (A) and 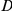 **(B).** Low spatial proximity values correspond to the fragments distant from each other, high values correspond to proximal fragments.(TIFF)

Figure S5
**Correlations of the spatial proximity values in the matrix** 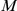 * with expression (A), histone modifications (B), DNA methylation (C), and DNAse sensitivity (D) differences. Symbols in B show the medians for different histone modifications. Other notations are as in Fig. 3.(TIFF)

Figure S6
**Correlations of the spatial proximity values in the matrix** 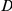 **with expression (A), histone modifications (B), DNA methylation (C), and DNAse sensitivity (D) differences. Symbols in B show the medians for different histone modifications.** Other notations are as in Fig. 3.(TIFF)

Figure S7 **Correlations of the spatial proximity values with the gene density. All notations are as in** **Fig. 3** **.**
(TIFF)

Figure S8 **Correlations of the spatial proximity values by compartments with expression, DNA methylation, DNAse sensitivity and various histone modification differences. AA denotes the pairs with both fragments in open chromatin compartment, BB, both fragments are in closed chromatin compartment; AB, fragments are in different compartments.** Other notations are as in Fig. 3.
(TIFF)

Figure S9 **Histone modifications and their differences within the cluster. DNA fragments clustered into 16 groups. All notations are as in** **Fig. 3** **.**
(TIFF)

Figure S10 **DNA fragments clustered into 4 groups.** (A) cluster size; (B) distances between all possible pairs of DNA fragments within the cluster; (C) distances between each DNA fragment from the cluster and each DNA fragment from the remaining set; (D-F) expression, DNA methylation, and DNAse sensitivity levels within the cluster; (G-I) expression, DNA methylation, and DNAse sensitivity differences within the cluster.
(TIFF)

Figure S11 **DNA fragments clustered into 8 groups.** (A) cluster size; (B) distances between all possible pairs of DNA fragments within the cluster; (C) distances between each DNA fragment from the cluster and each DNA fragment from the remaining set; (D-F) expression, DNA methylation, and DNAse sensitivity levels within the cluster; (G-I) expression, DNA methylation, and DNAse sensitivity differences within the cluster.
(TIFF)

Figure S12 **DNA fragments clustered into 32 groups.** (A) cluster size; (B) distances between all possible pairs of DNA fragments within the cluster; (C) distances between each DNA fragment from the cluster and each DNA fragment from the remaining set; (D-F) expression, DNA methylation, and DNAse sensitivity levels within the cluster; (G-I) expression, DNA methylation, and DNAse sensitivity differences within the cluster.
(TIFF)

Figure S13 **DNA fragments clustered into 64 groups. (A) cluster size; (B) distances between all possible pairs of DNA fragments within the cluster; (C) distances between each DNA fragment from the cluster and each DNA fragment from the remaining set; (D-F) expression, DNA methylation, and DNAse sensitivity levels within the cluster; (G-I) expression, DNA methylation, and DNAse sensitivity differences within the cluster.**
(TIFF)

Figure S14 **The linear proximity values within the clusters.** The linear proximity values were calculated as 1 divided by the distance between the centers of interacting fragments in Mbases if the fragments were located on the same chromosome, and were equal 0 otherwise. Means represented by dots, standard deviations, by lines. The upper row of figures represents corresponding spatial proximity values, for comparison. The whisker boxes are as in Fig. 3.
(TIFF)

Figure S15 **The root mean squared error (RMSE) of the spatial proximity prediction for the pairs of genome fragments originating at the open chromatin compartment (AA).** All notations are as in Fig. 9.
(TIFF)

Figure S16 **The root mean squared error (RMSE) of the spatial proximity prediction for the pairs of genome fragments originating at the closed chromatin compartment (BB).** All notations are as in Fig. 9.
(TIFF)

Figure S17 **The root mean squared error (RMSE) of the spatial proximity prediction for the pairs of genome fragments originating at different compartments (AB).** All notations are as in Fig. 9.
(TIFF)

Figure S18 **Spatial proximity values plotted against sums of expression values.** Markers represent aggregated sample values, the line visualizes the regression model.
(TIFF)

Figure S19 **Spatial proximity values plotted against sums of DNAse sensitivity values.** Markers represent aggregated sample values, the line visualizes the regression model.
(TIFF)

Figure S20 **Spatial proximity values plotted against sums of methylation state values.** Markers represent aggregated sample values, the line visualizes the regression model.
(TIFF)

Figure S21 **Spatial proximity values plotted against sums of histone modifications values.** Markers represent aggregated sample values.
(TIFF)

Figure S22 **Spatial proximity values plotted against differences of expression values.** Markers represent aggregated sample values, the line visualizes the regression model.
(TIFF)

Figure S23 **Spatial proximity values plotted against differences of DNAse sensitivity values.** Markers represent aggregated sample values, the line visualizes the regression model.
(TIFF)

Figure S24 **Spatial proximity values plotted against differences of methylation state values.** Markers represent aggregated sample values, the line visualizes the regression model.
(TIFF)

Figure S25 **Spatial proximity values plotted against differences of histone modifications values. Markers represent aggregated sample values.**
(TIFF)

Table S1 The RMSE of regression models, which use one feature separately to predict spatial proximity, compared to the RMSE of the algorithm which uses training set mean as the predicted value. The significance of the difference between each feature-based model and the mean-based algorithm was estimated by p-values of paired, two-sample, two-tailed T-test with the Bonferroni correction, which are shown right to the corresponding model errors. Bold font shows features, for which the regression models have larger error than the mean-based algorithm. Italic font shows non-significant differences.
(PDF)

Table S2 The list of DNA fragments clustered into 4, 8, 16, 32, and 64 groups by the spatial proximity.
(XLS)
