# Supplementary figures and images for: Spatial Proximity and Similarity of the Epigenetic State of Genome Domains
Source: PLoS One. 2012 Apr 4;7(4):e33947. doi: 10.1371/journal.pone.0033947 (PMC3319547; doi:10.1371/journal.pone.0033947)

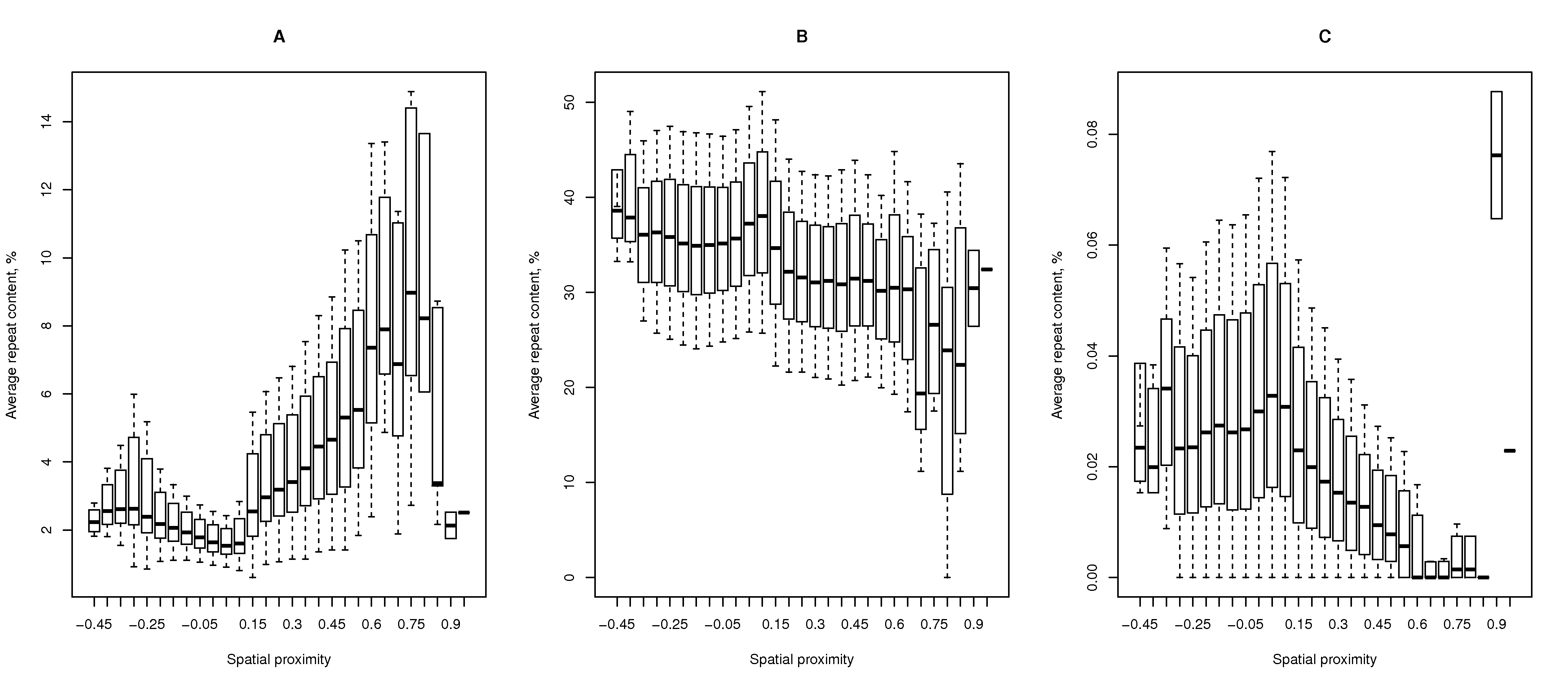

Supplement: Figure S1 — The average repeat content in 29 considered intervals of the spatial proximity in the the genome-wide correlation matrix C. (A) Simple repeats. (B) Nested repeats. (C) Exapted repeats. (TIFF) [file pone.0033947.s001.tiff]

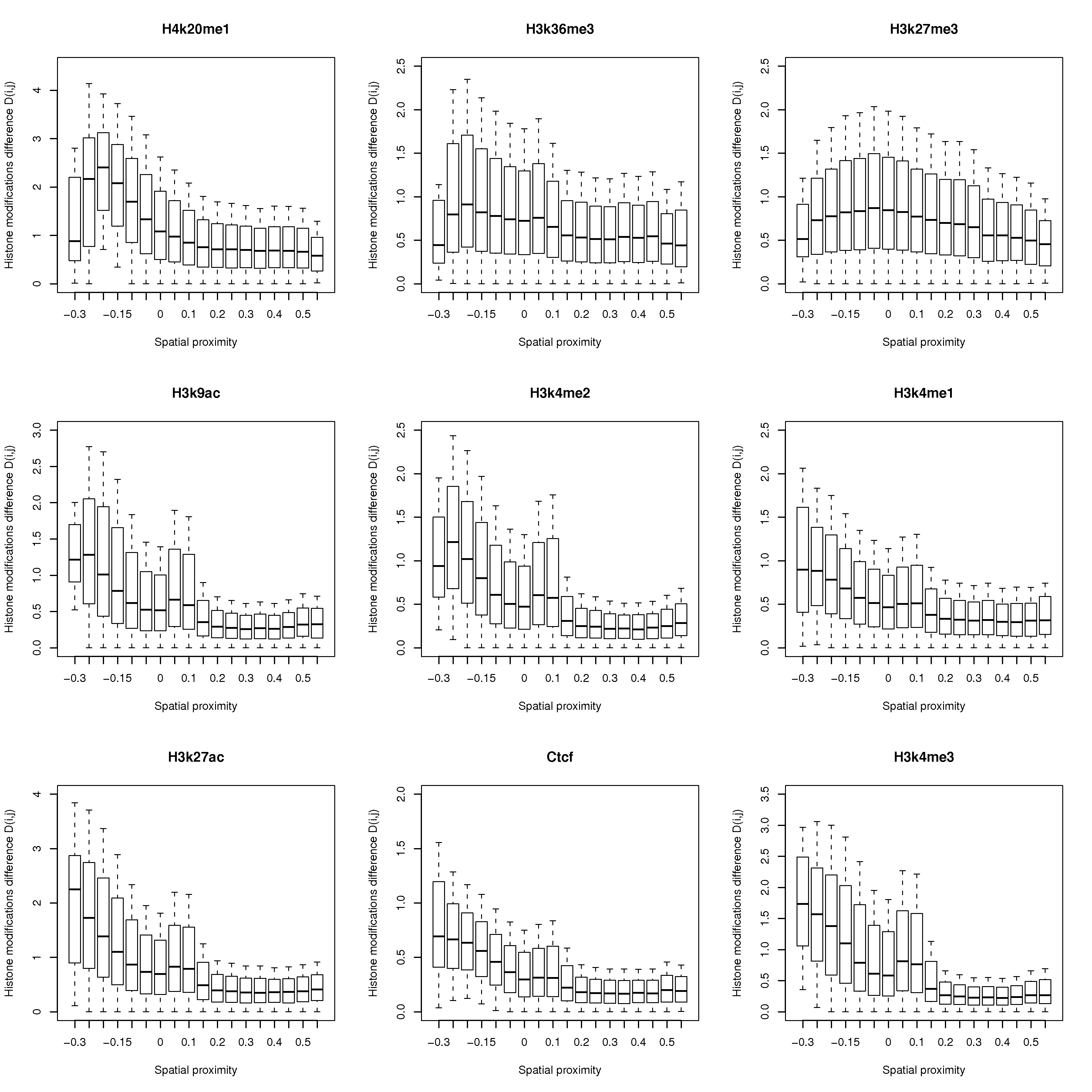

Supplement: Figure S2 — Correlations of the spatial proximity values with histone modifications differences. All notations are as in Fig. 3 . (TIFF) [file pone.0033947.s002.tiff]

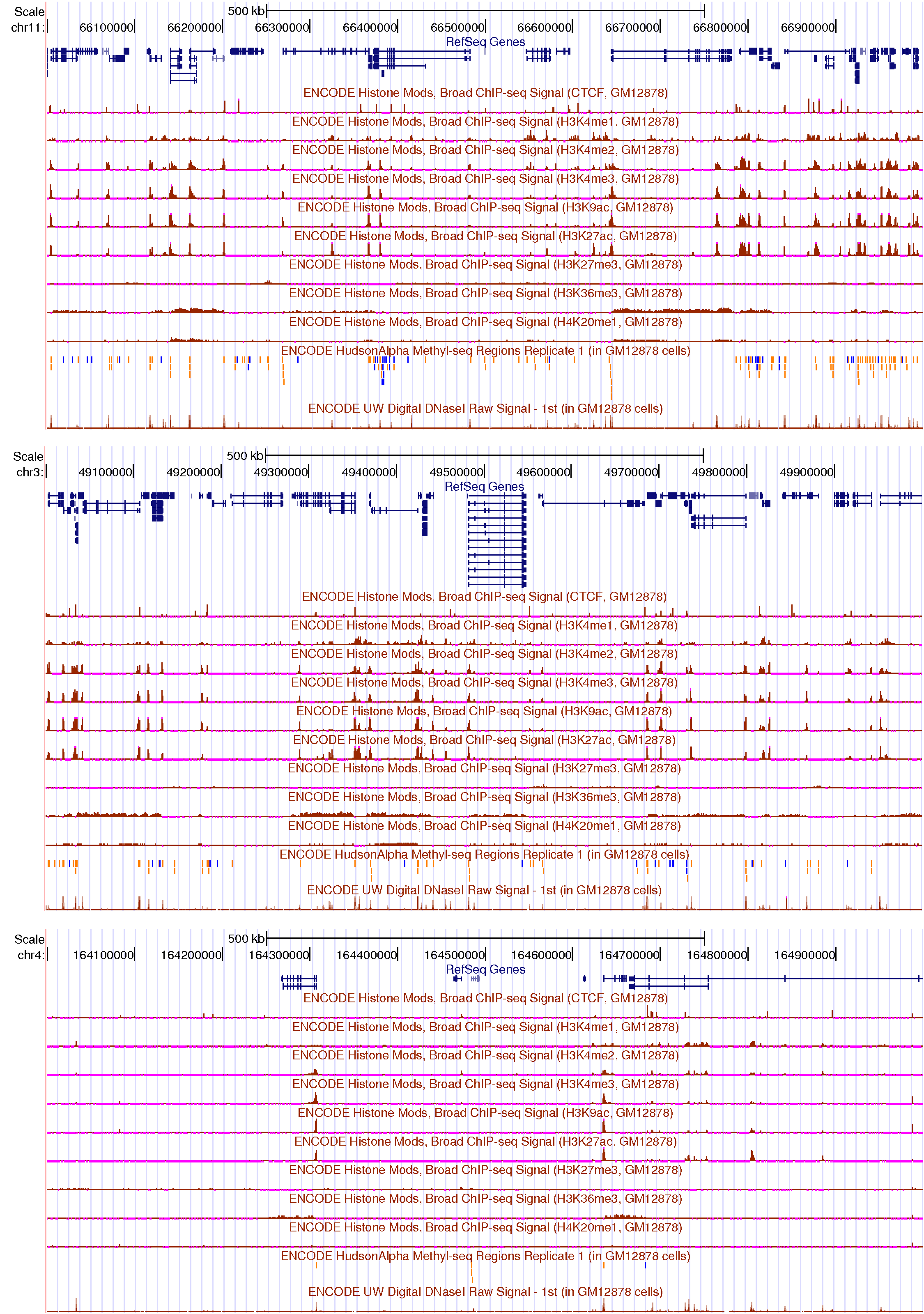

Supplement: Figure S3 — Examples of spatially proximal (chr3:49000000–49999999 and chr11:66000000–66999999, spatial proximity = 0.54) and spatially distant (chr3:49000000–49999999 and chr4:164000000–164999999, spatial proximity = −0.25; chr4:164000000–164999999 and chr11:66000000–66999999, spatial proximity = −0.22) fragments. Spatially proximal fragments have similar epigenetic features, while spatially distant have rather different ones. (TIFF) [file pone.0033947.s003.tiff]

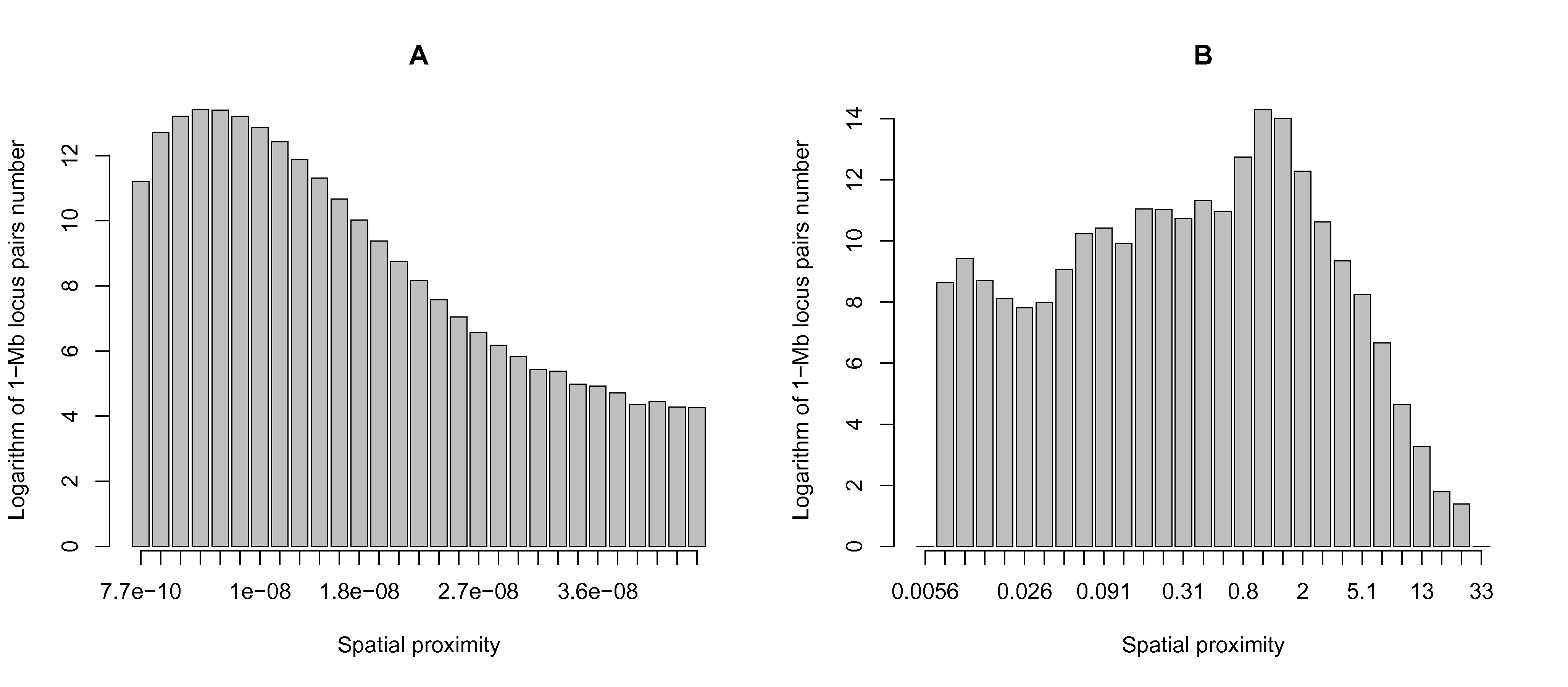

Supplement: Figure S4 — Histograms of the number of pairs of the interacting genome fragments originating at different chromosomes in the human genome-wide spatial proximity matrices * (A) and (B). Low spatial proximity values correspond to the fragments distant from each other, high values correspond to proximal fragments. (TIFF) [file pone.0033947.s004.tiff]

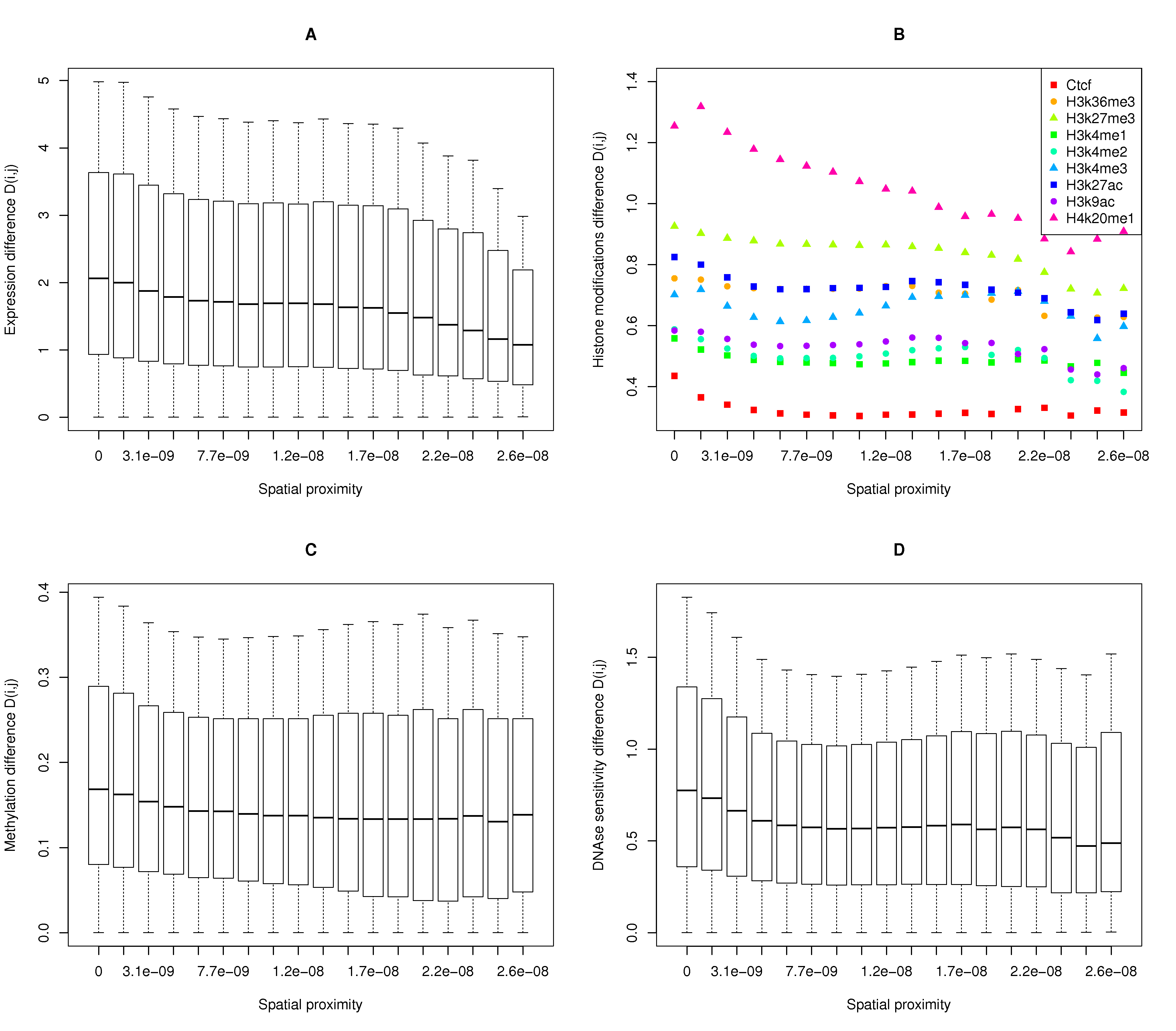

Supplement: Figure S5 — Correlations of the spatial proximity values in the matrix * with expression (A), histone modifications (B), DNA methylation (C), and DNAse sensitivity (D) differences. Symbols in B show the medians for different histone modifications. Other notations are as in Fig. 3. (TIFF) [file pone.0033947.s005.tiff]

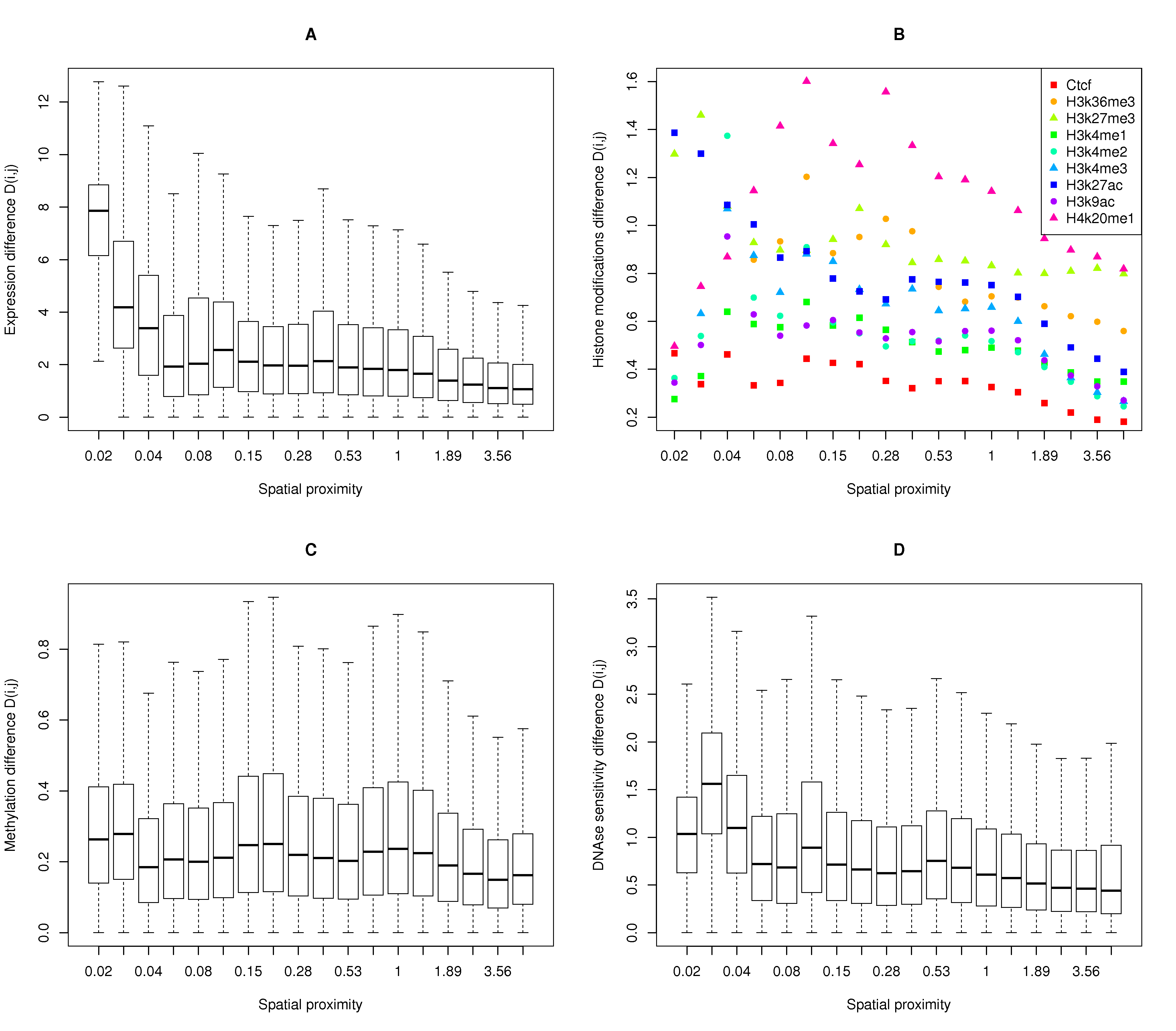

Supplement: Figure S6 — Correlations of the spatial proximity values in the matrix with expression (A), histone modifications (B), DNA methylation (C), and DNAse sensitivity (D) differences. Symbols in B show the medians for different histone modifications. Other notations are as in Fig. 3. (TIFF) [file pone.0033947.s006.tiff]

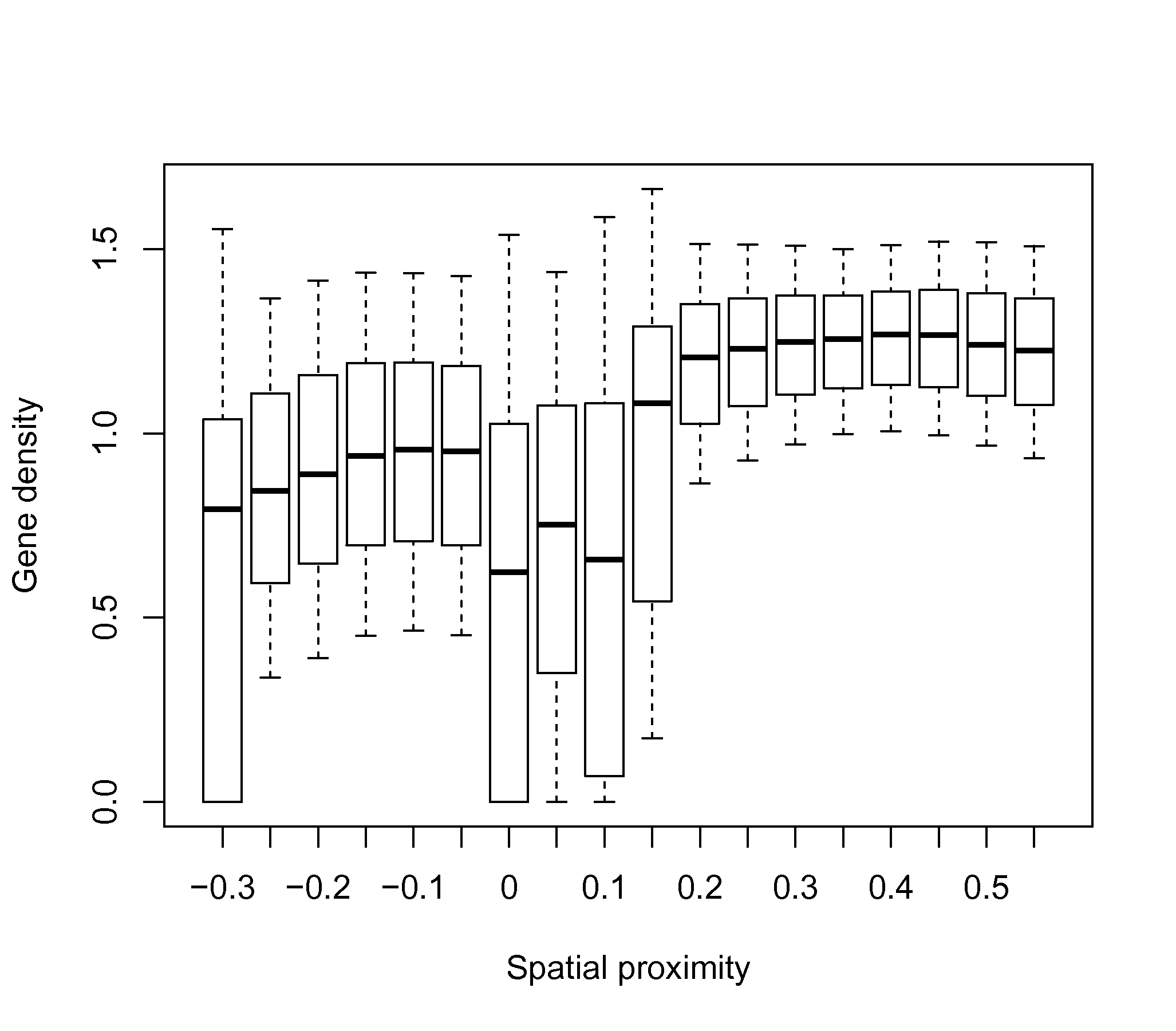

Supplement: Figure S7 — Correlations of the spatial proximity values with the gene density. All notations are as in Fig. 3 . (TIFF) [file pone.0033947.s007.tiff]

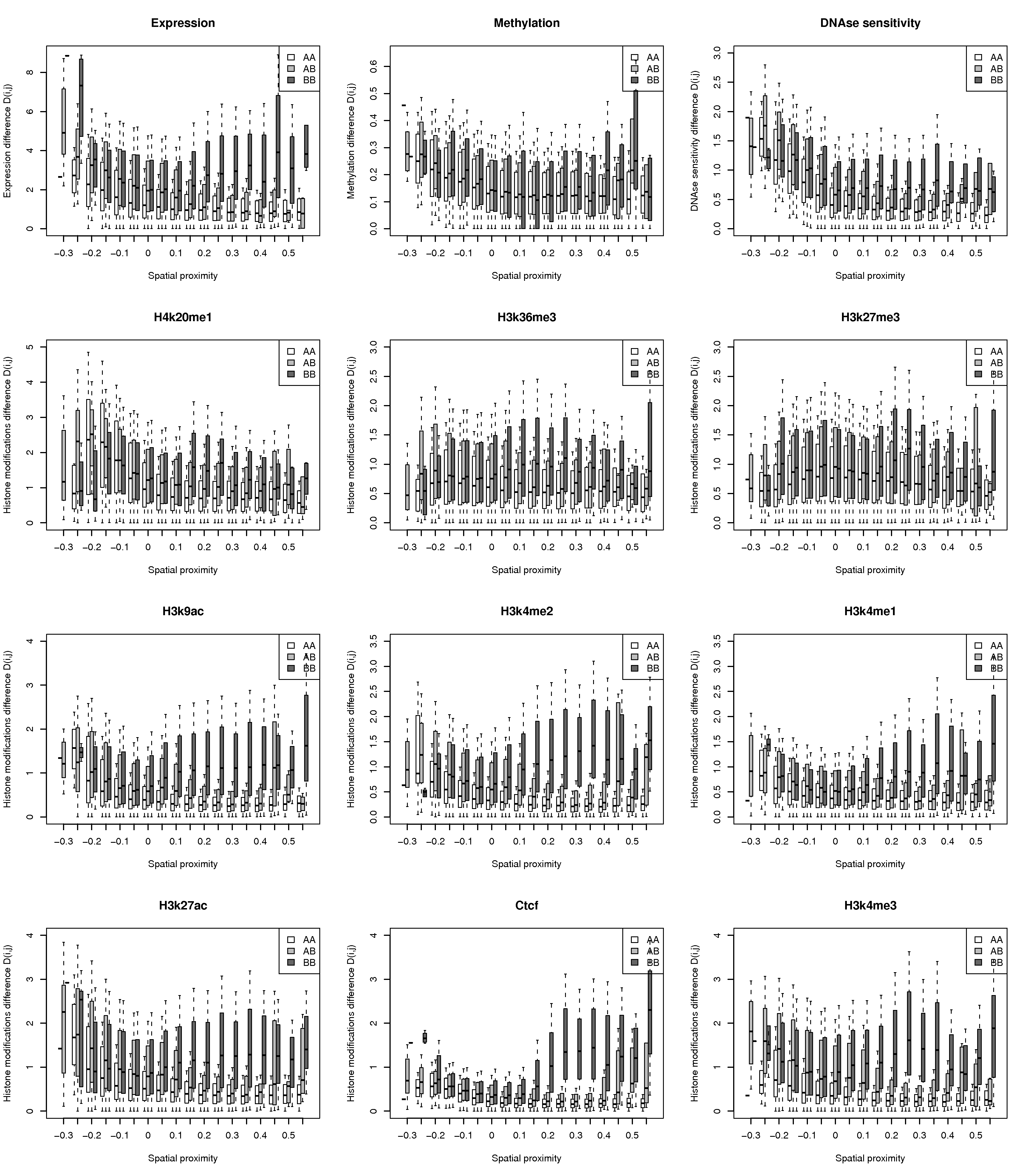

Supplement: Figure S8 — Correlations of the spatial proximity values by compartments with expression, DNA methylation, DNAse sensitivity and various histone modification differences. AA denotes the pairs with both fragments in open chromatin compartment, BB, both fragments are in closed chromatin compartment; AB, fragments are in different compartments. Other notations are as in Fig. 3. (TIFF) [file pone.0033947.s008.tiff]

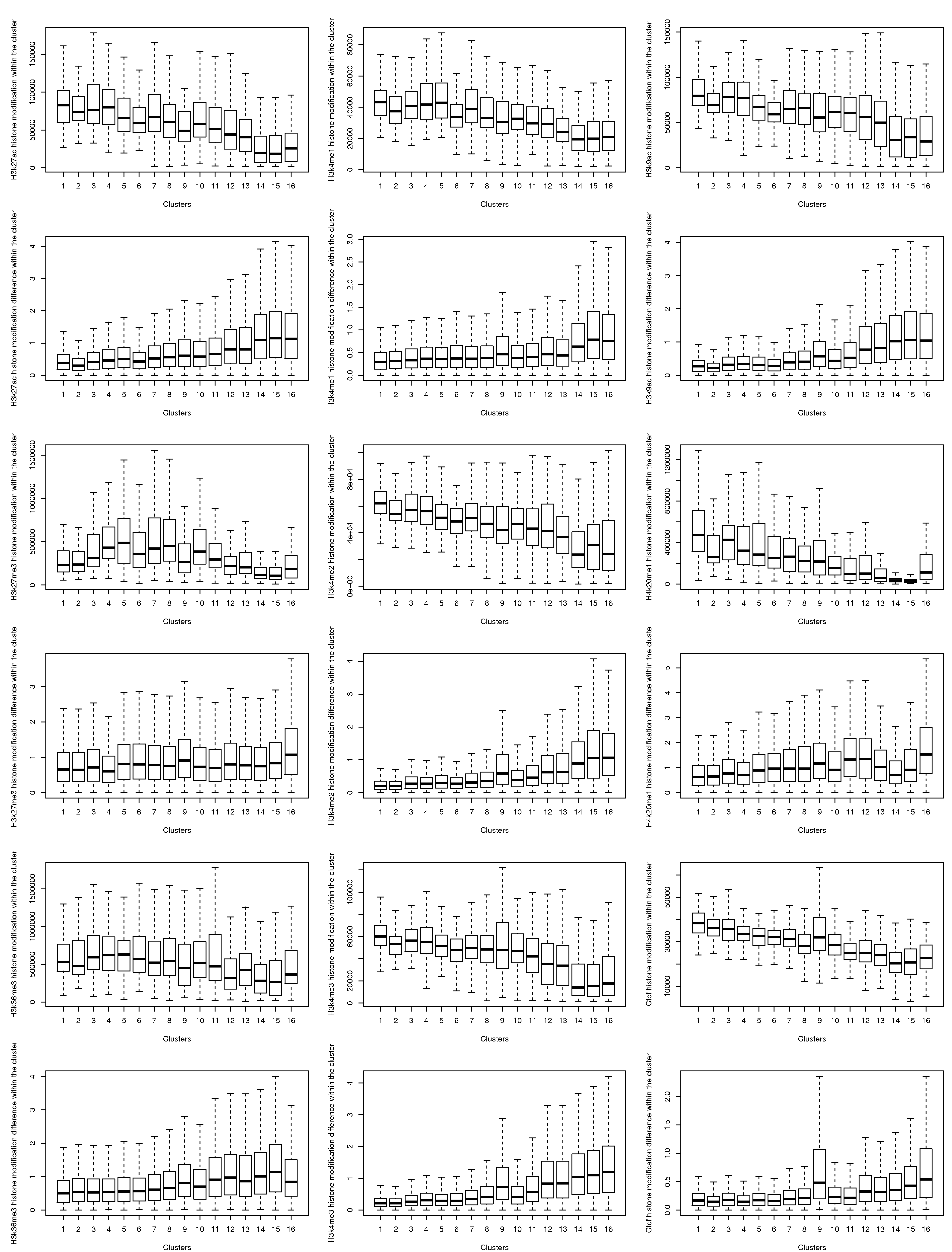

Supplement: Figure S9 — Histone modifications and their differences within the cluster. DNA fragments clustered into 16 groups. All notations are as in Fig. 3 . (TIFF) [file pone.0033947.s009.tiff]

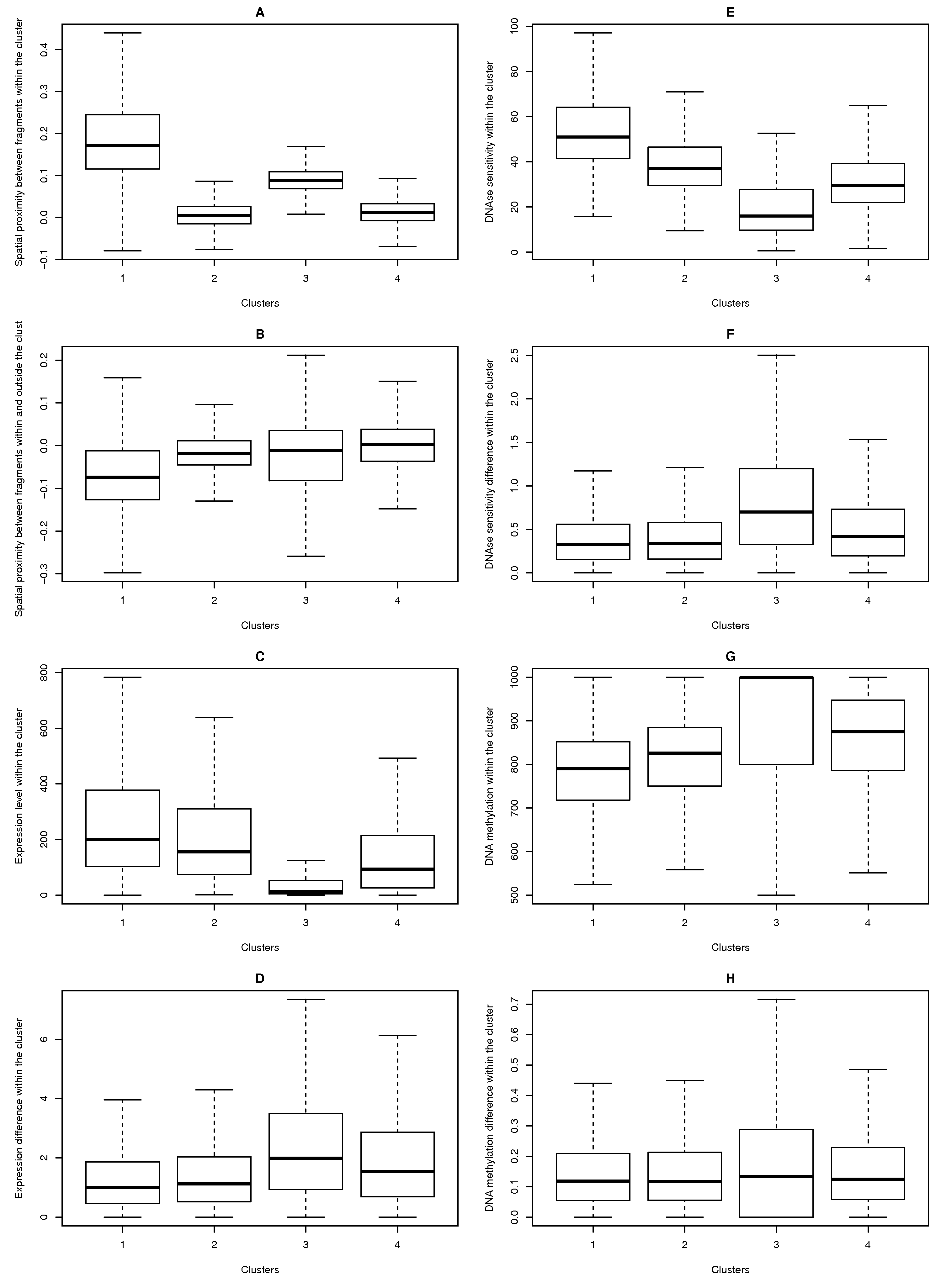

Supplement: Figure S10 — DNA fragments clustered into 4 groups. (A) cluster size; (B) distances between all possible pairs of DNA fragments within the cluster; (C) distances between each DNA fragment from the cluster and each DNA fragment from the remaining set; (D-F) expression, DNA methylation, and DNAse sensitivity levels within the cluster; (G-I) expression, DNA methylation, and DNAse sensitivity differences within the cluster. (TIFF) [file pone.0033947.s010.tiff]

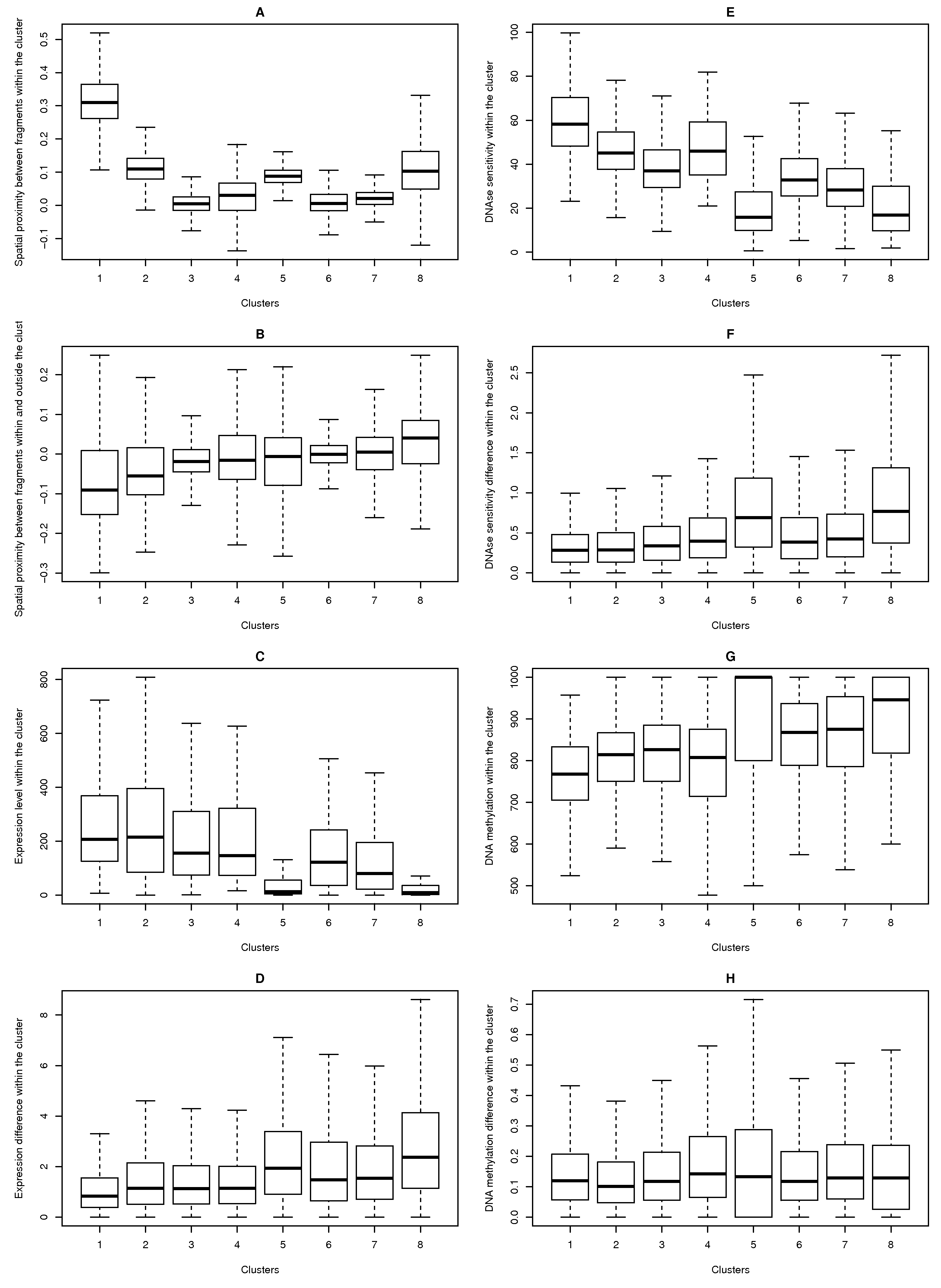

Supplement: Figure S11 — DNA fragments clustered into 8 groups. (A) cluster size; (B) distances between all possible pairs of DNA fragments within the cluster; (C) distances between each DNA fragment from the cluster and each DNA fragment from the remaining set; (D-F) expression, DNA methylation, and DNAse sensitivity levels within the cluster; (G-I) expression, DNA methylation, and DNAse sensitivity differences within the cluster. (TIFF) [file pone.0033947.s011.tiff]

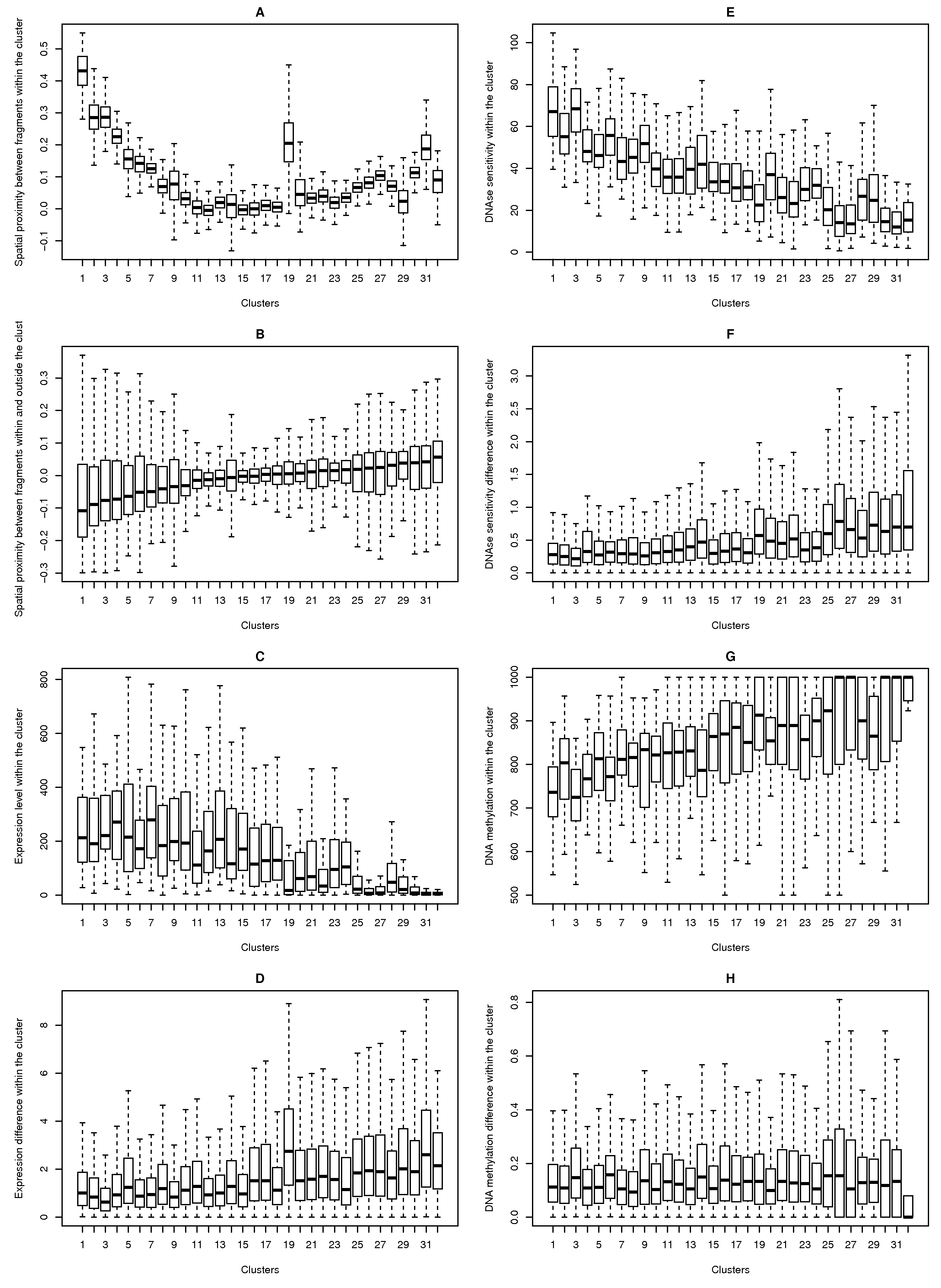

Supplement: Figure S12 — DNA fragments clustered into 32 groups. (A) cluster size; (B) distances between all possible pairs of DNA fragments within the cluster; (C) distances between each DNA fragment from the cluster and each DNA fragment from the remaining set; (D-F) expression, DNA methylation, and DNAse sensitivity levels within the cluster; (G-I) expression, DNA methylation, and DNAse sensitivity differences within the cluster. (TIFF) [file pone.0033947.s012.tiff]

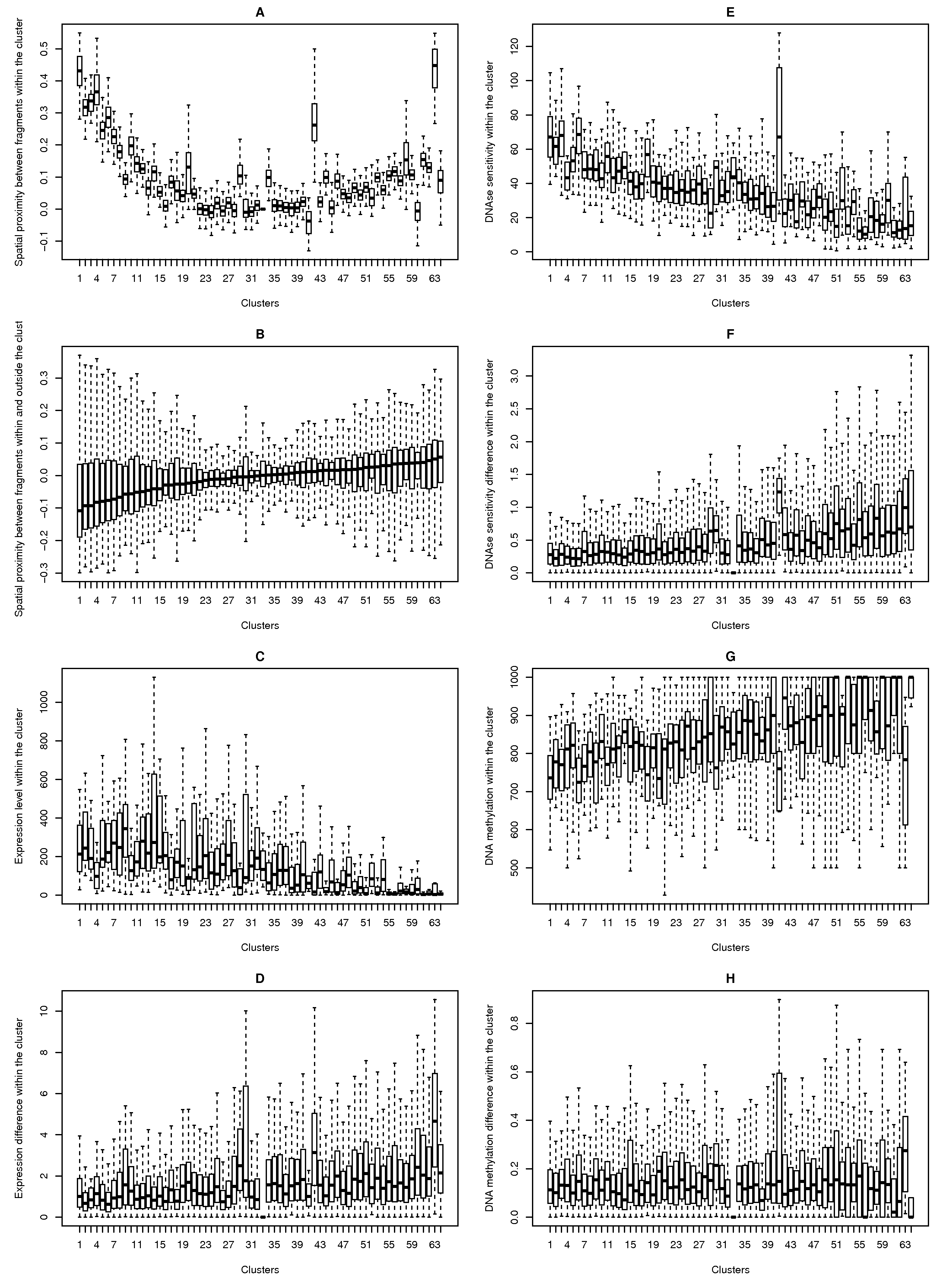

Supplement: Figure S13 — DNA fragments clustered into 64 groups. (A) cluster size; (B) distances between all possible pairs of DNA fragments within the cluster; (C) distances between each DNA fragment from the cluster and each DNA fragment from the remaining set; (D-F) expression, DNA methylation, and DNAse sensitivity levels within the cluster; (G-I) expression, DNA methylation, and DNAse sensitivity differences within the cluster. (TIFF) [file pone.0033947.s013.tiff]

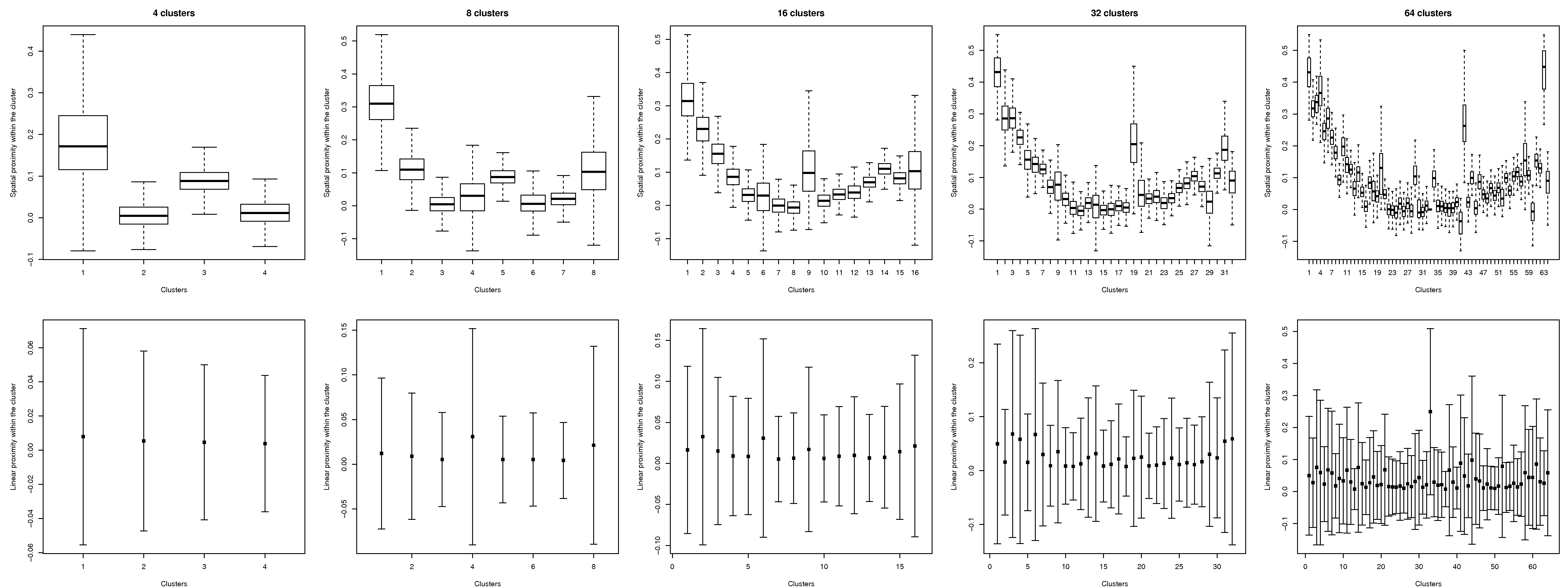

Supplement: Figure S14 — The linear proximity values within the clusters. The linear proximity values were calculated as 1 divided by the distance between the centers of interacting fragments in Mbases if the fragments were located on the same chromosome, and were equal 0 otherwise. Means represented by dots, standard deviations, by lines. The upper row of figures represents corresponding spatial proximity values, for comparison. The whisker boxes are as in Fig. 3. (TIFF) [file pone.0033947.s014.tiff]

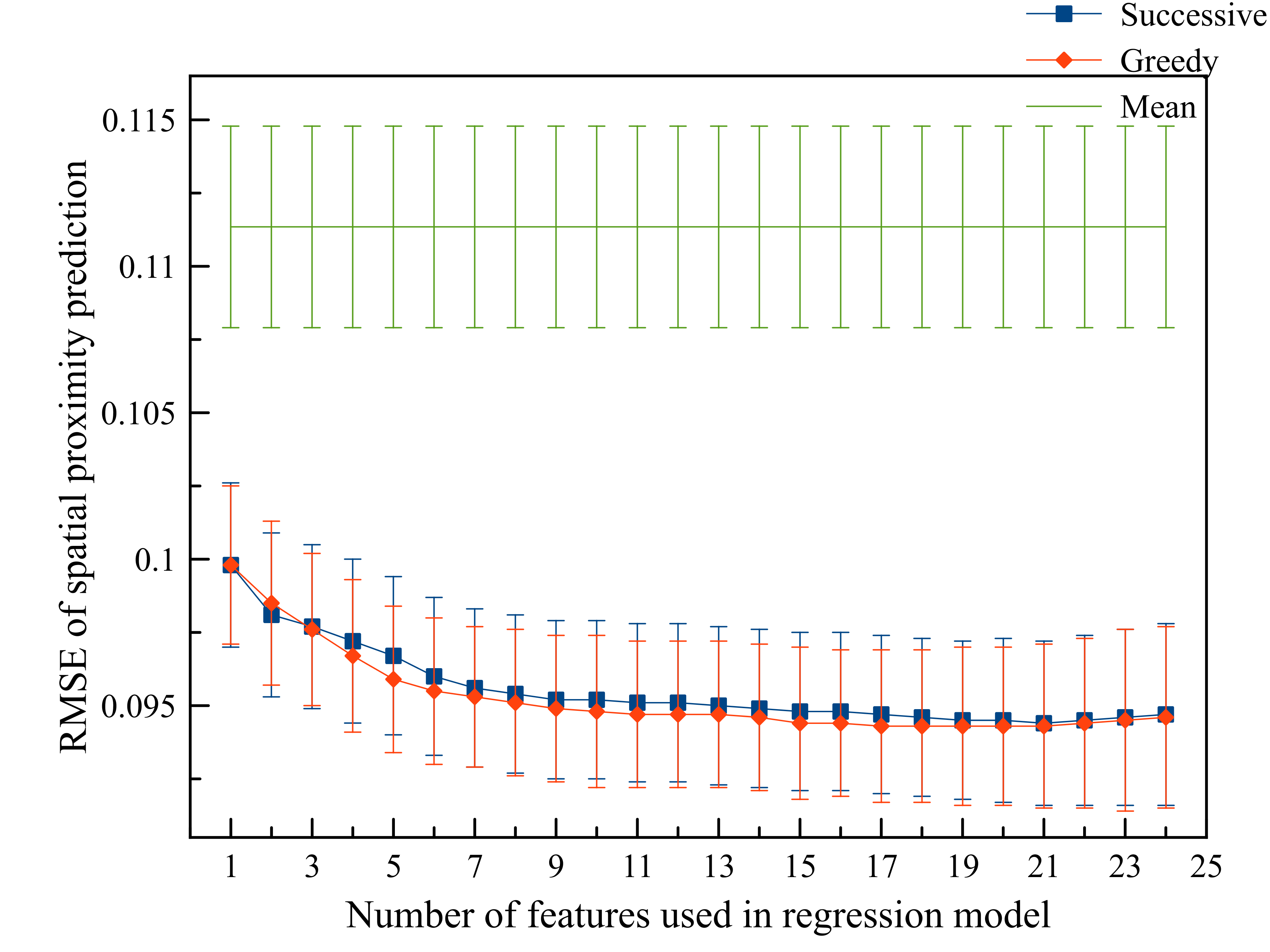

Supplement: Figure S15 — The root mean squared error (RMSE) of the spatial proximity prediction for the pairs of genome fragments originating at the open chromatin compartment (AA). All notations are as in Fig. 9. (TIFF) [file pone.0033947.s015.tiff]

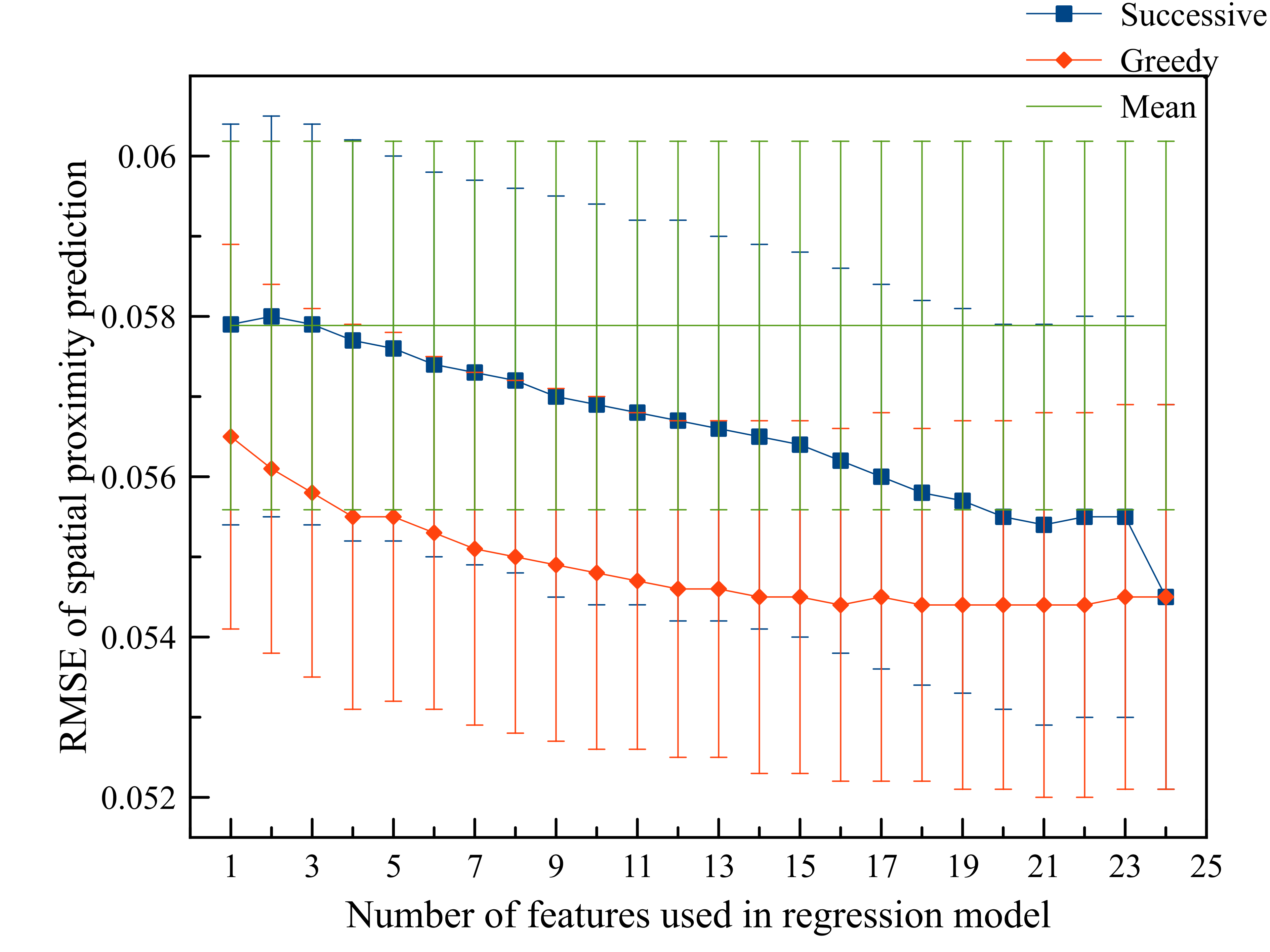

Supplement: Figure S16 — The root mean squared error (RMSE) of the spatial proximity prediction for the pairs of genome fragments originating at the closed chromatin compartment (BB). All notations are as in Fig. 9. (TIFF) [file pone.0033947.s016.tiff]

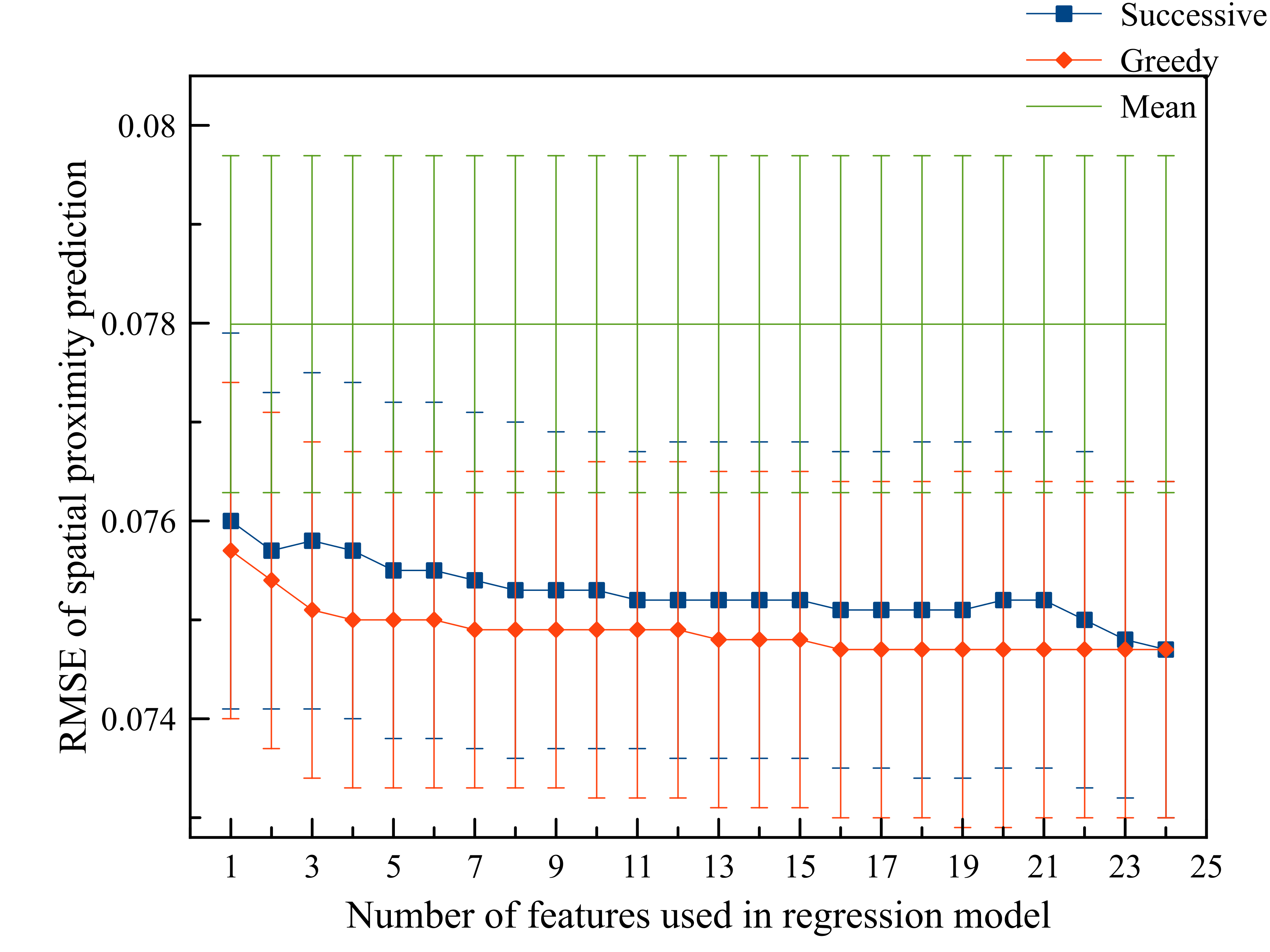

Supplement: Figure S17 — The root mean squared error (RMSE) of the spatial proximity prediction for the pairs of genome fragments originating at different compartments (AB). All notations are as in Fig. 9. (TIFF) [file pone.0033947.s017.tiff]

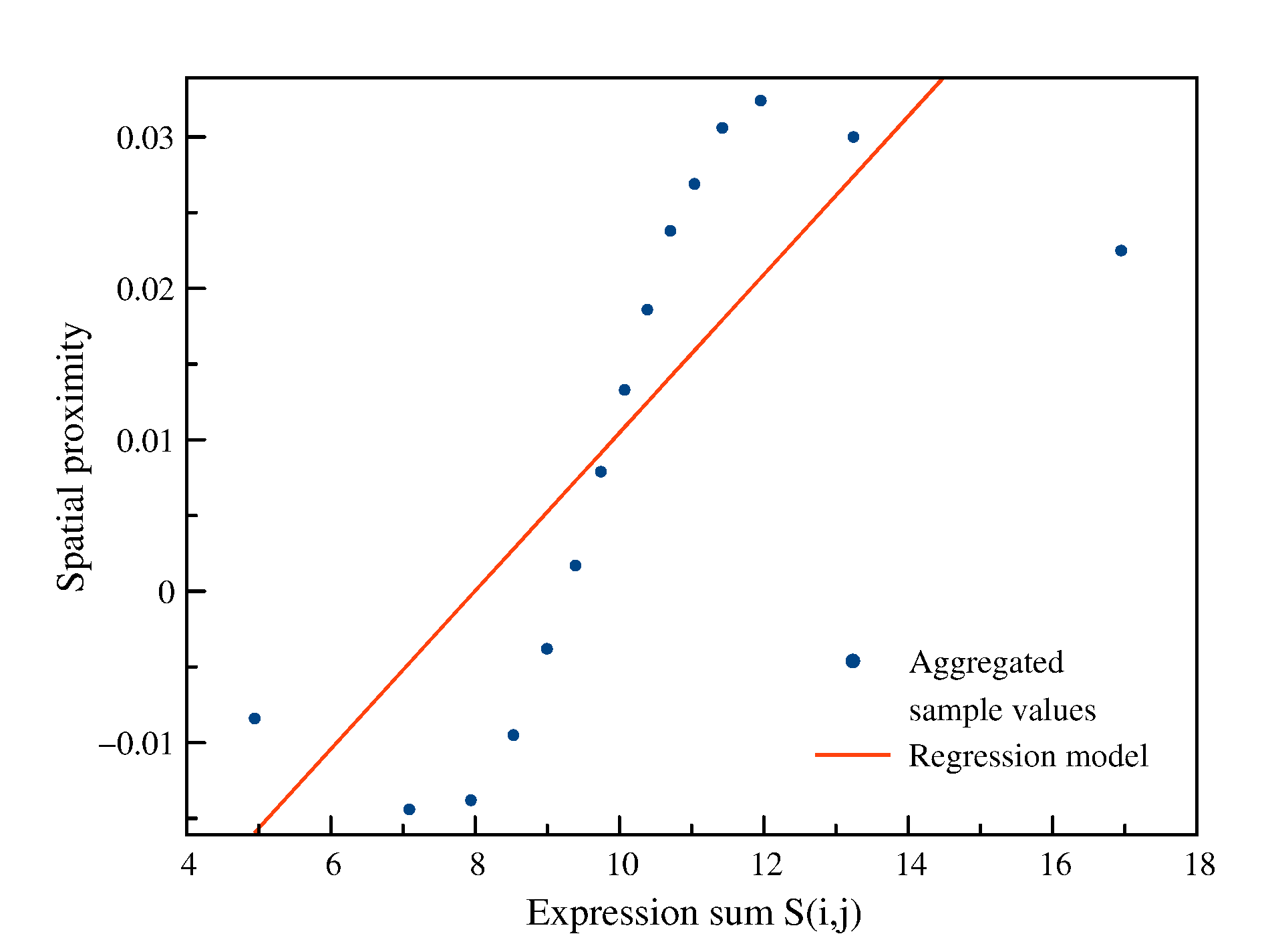

Supplement: Figure S18 — Spatial proximity values plotted against sums of expression values. Markers represent aggregated sample values, the line visualizes the regression model. (TIFF) [file pone.0033947.s018.tiff]

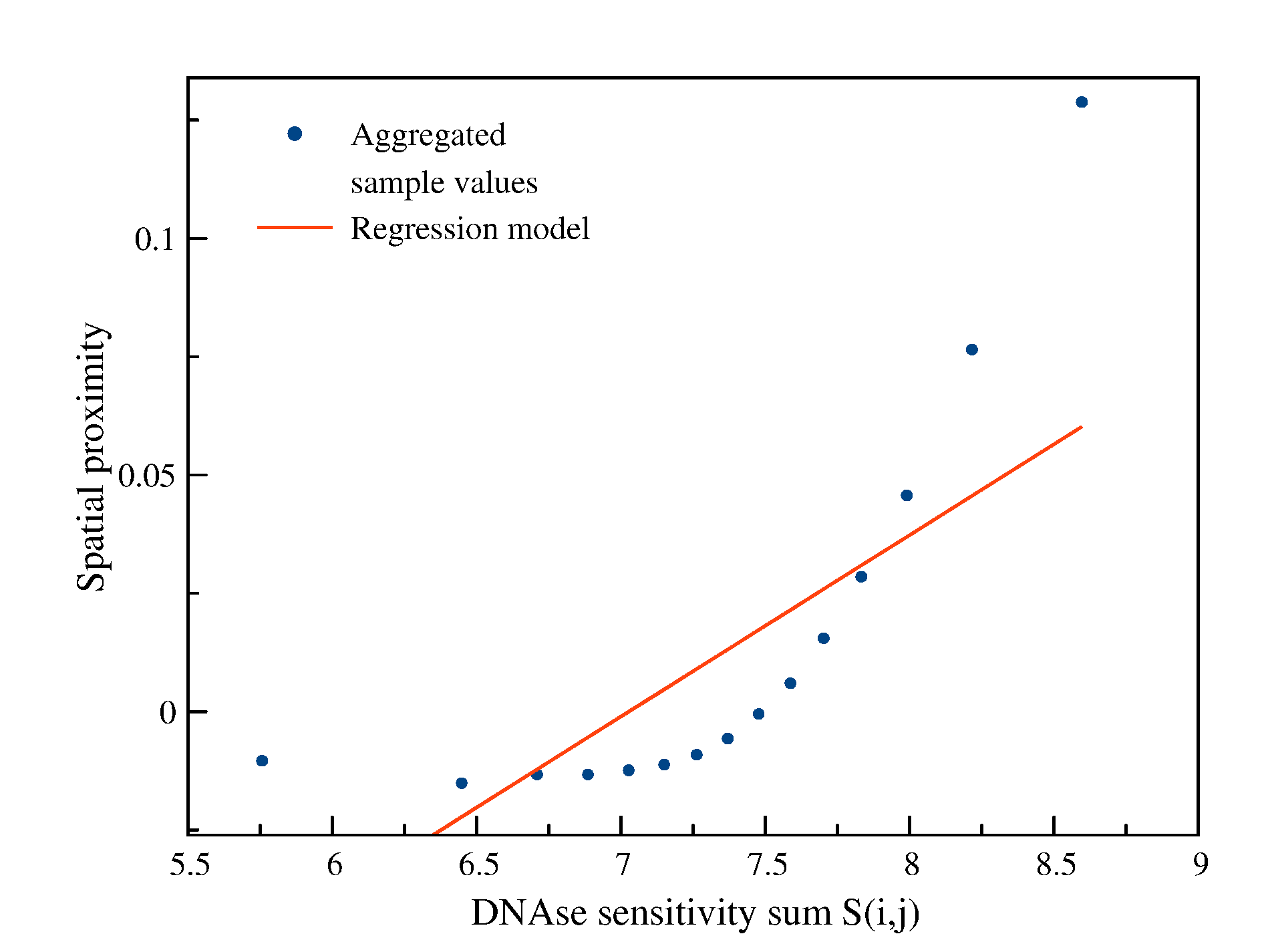

Supplement: Figure S19 — Spatial proximity values plotted against sums of DNAse sensitivity values. Markers represent aggregated sample values, the line visualizes the regression model. (TIFF) [file pone.0033947.s019.tiff]

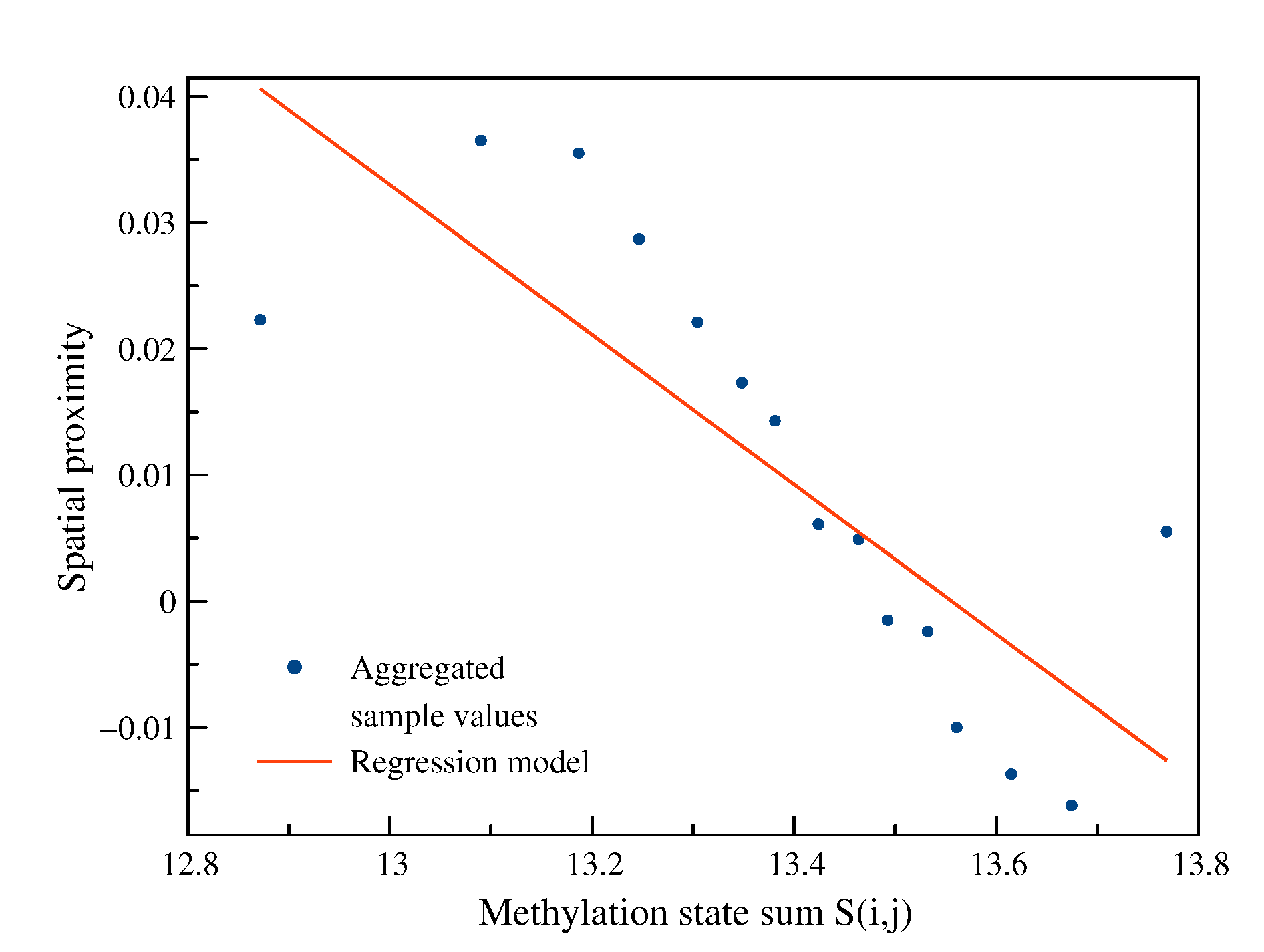

Supplement: Figure S20 — Spatial proximity values plotted against sums of methylation state values. Markers represent aggregated sample values, the line visualizes the regression model. (TIFF) [file pone.0033947.s020.tiff]

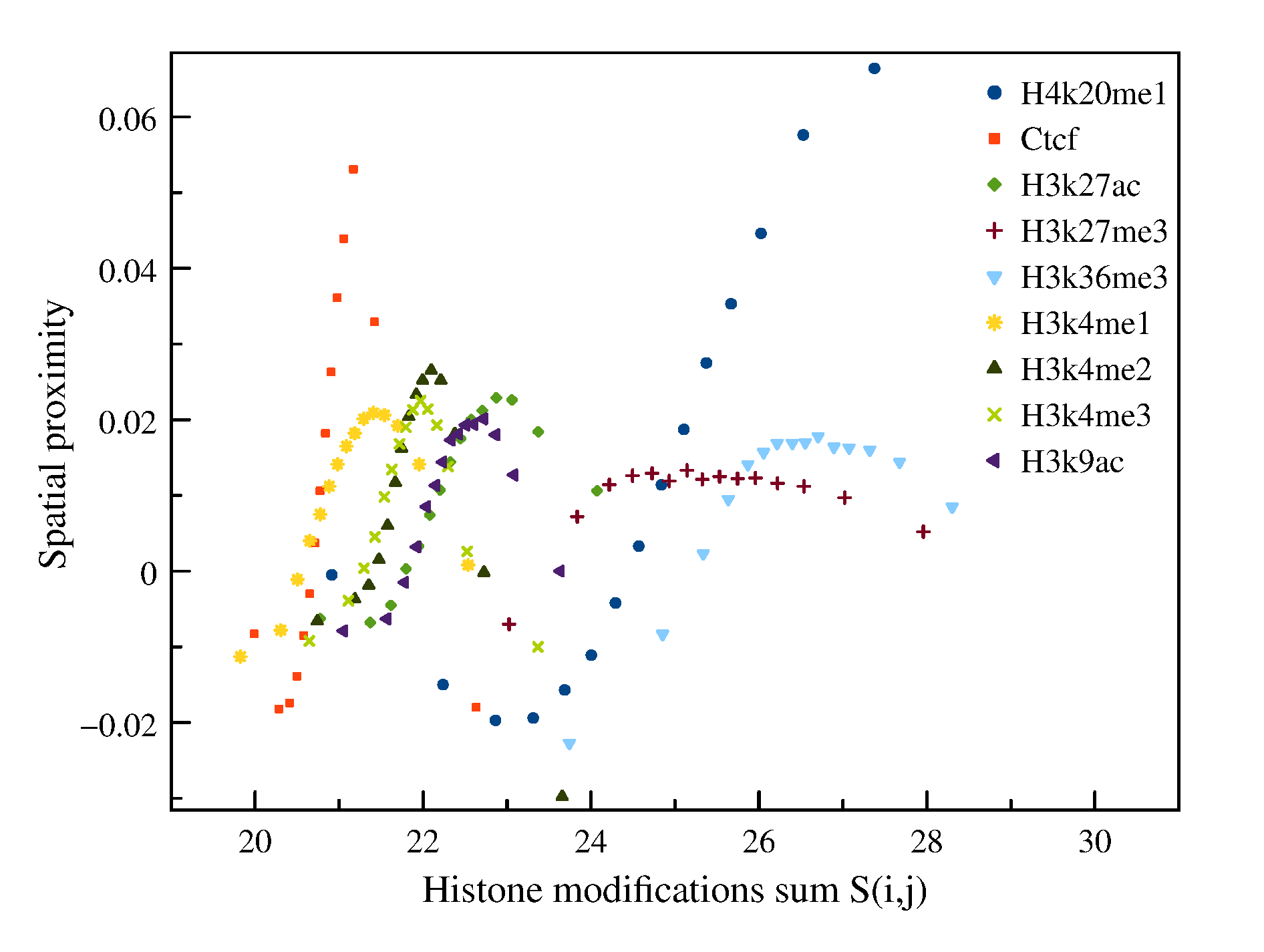

Supplement: Figure S21 — Spatial proximity values plotted against sums of histone modifications values. Markers represent aggregated sample values. (TIFF) [file pone.0033947.s021.tiff]

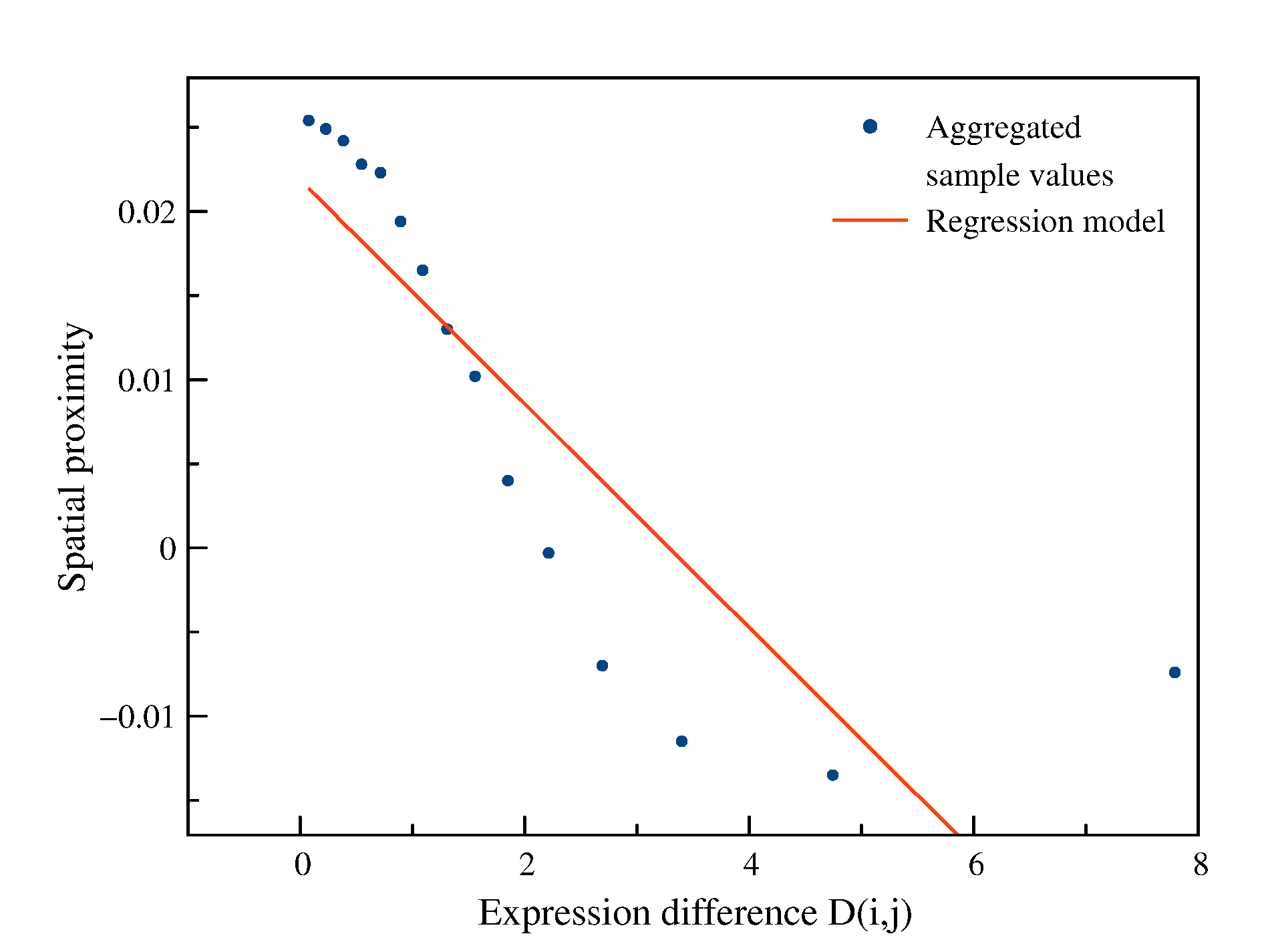

Supplement: Figure S22 — Spatial proximity values plotted against differences of expression values. Markers represent aggregated sample values, the line visualizes the regression model. (TIFF) [file pone.0033947.s022.tiff]

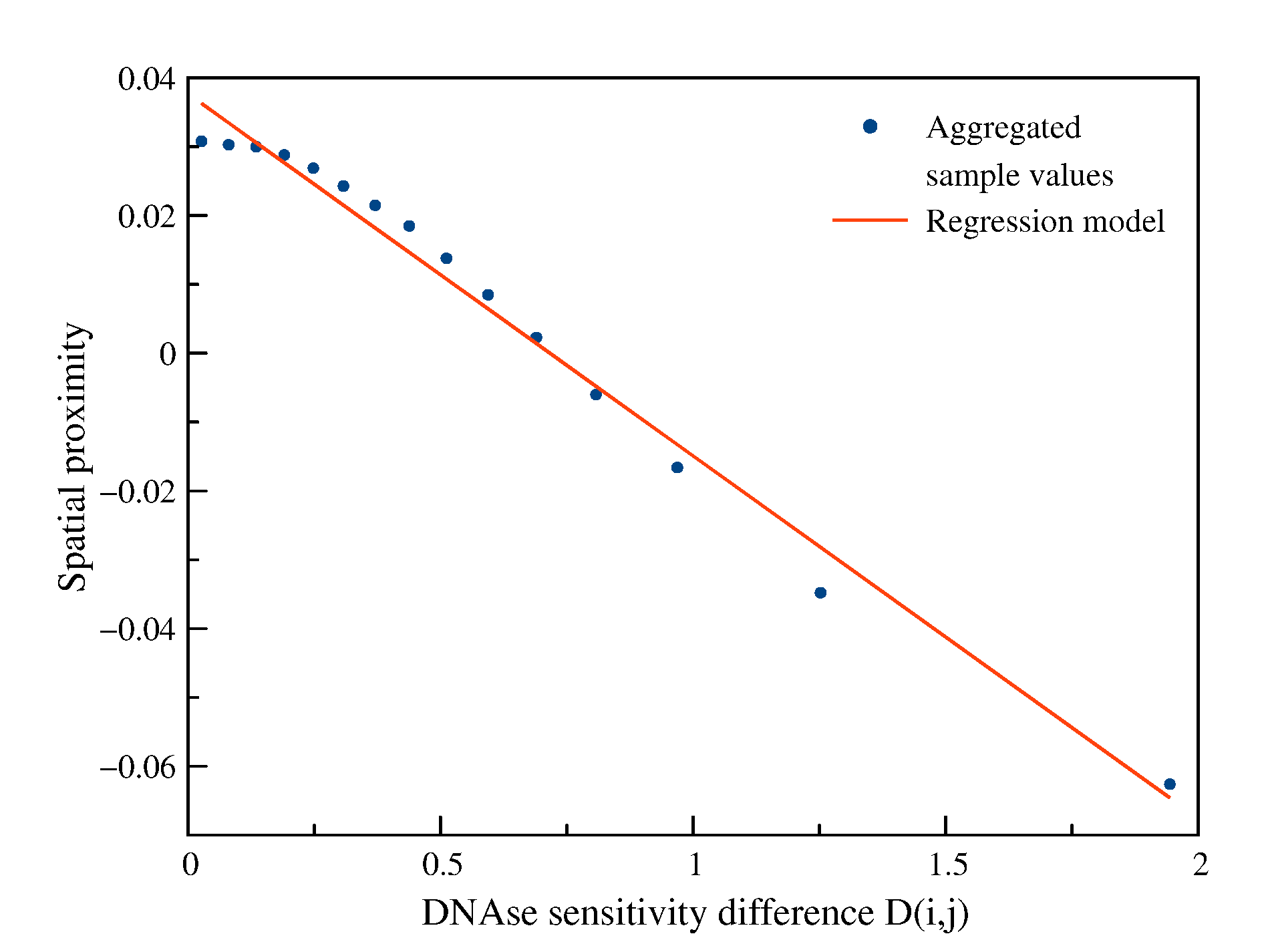

Supplement: Figure S23 — Spatial proximity values plotted against differences of DNAse sensitivity values. Markers represent aggregated sample values, the line visualizes the regression model. (TIFF) [file pone.0033947.s023.tiff]

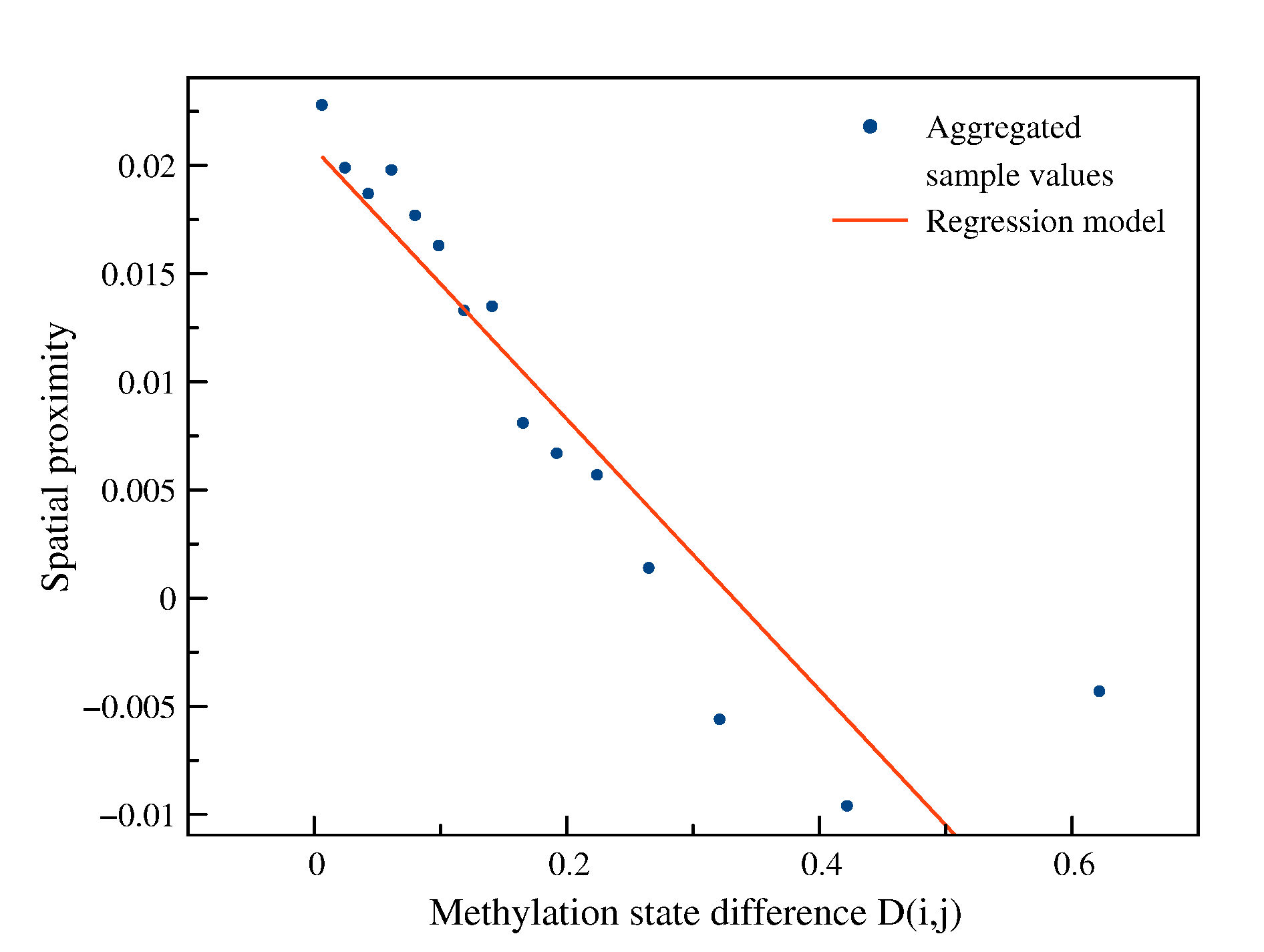

Supplement: Figure S24 — Spatial proximity values plotted against differences of methylation state values. Markers represent aggregated sample values, the line visualizes the regression model. (TIFF) [file pone.0033947.s024.tiff]

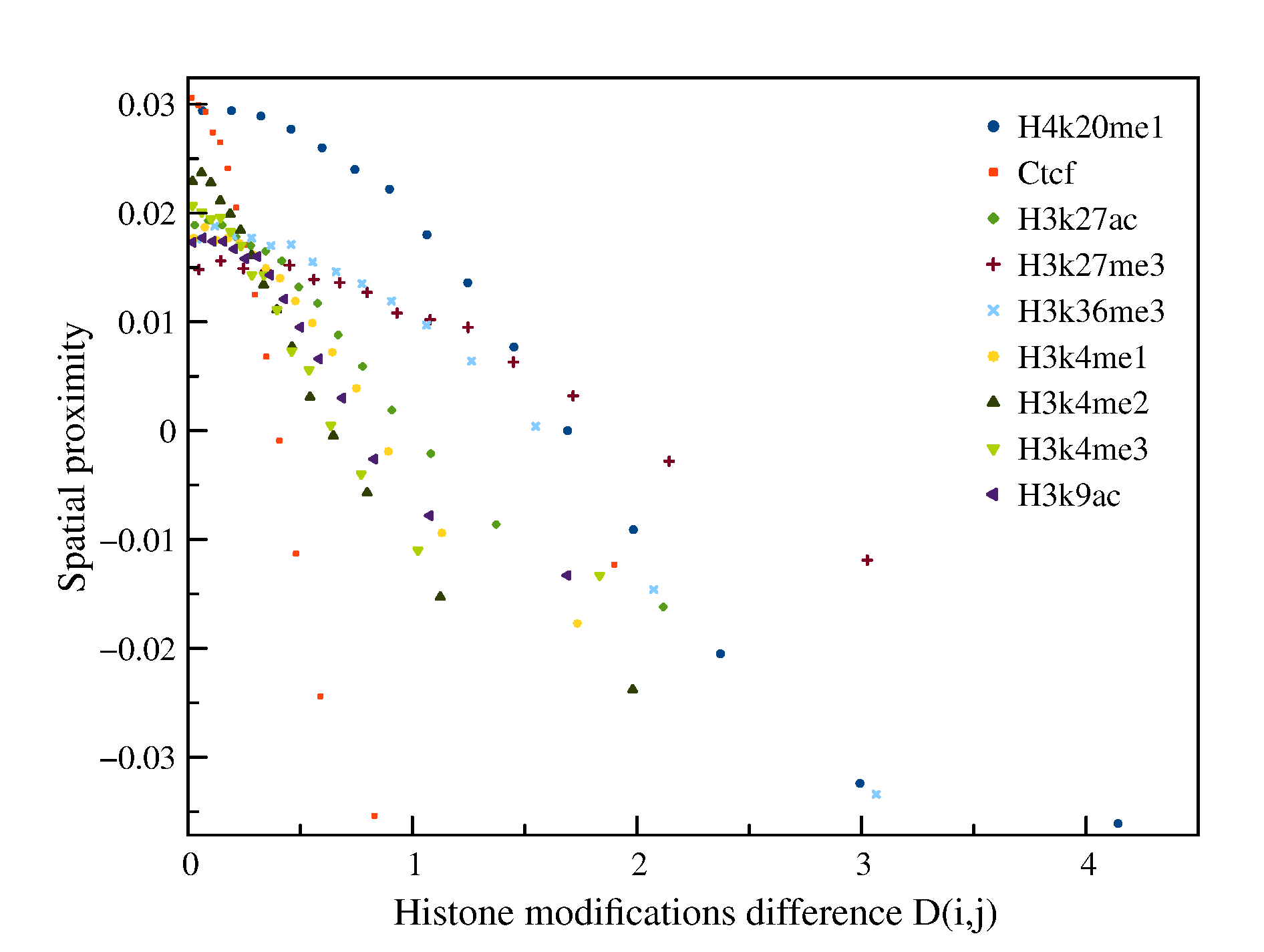

Supplement: Figure S25 — Spatial proximity values plotted against differences of histone modifications values. Markers represent aggregated sample values. (TIFF) [file pone.0033947.s025.tiff]
