# Supplementary material for: Spatial Proximity and Similarity of the Epigenetic State of Genome Domains
Source: PLoS One. 2012 Apr 4;7(4):e33947. doi: 10.1371/journal.pone.0033947 (PMC3319547; doi:10.1371/journal.pone.0033947)
Supplement: Table S1 — The RMSE of regression models, which use one feature separately to predict spatial proximity, compared to the RMSE of the algorithm which uses training set mean as the predicted value. The significance of the difference between each feature-based model and the mean-based algorithm was estimated by p-values of paired, two-sample, two-tailed T-test with the Bonferroni correction, which are shown right to the corresponding model errors. Bold font shows features, for which the regression models have larger error than the mean-based algorithm. Italic font shows non-significant differences. (PDF) [file pone.0033947.s026.pdf]

Feature	Sum	P-value	Difference	P-value
Expression	9.97E-02	1.05E-121	9.99E-02	3.65E-104
DNAse sensitivity	9.71E-02	2.12E-161	9.78E-02	2.55E-166
Methylation	9.96E-02	1.16E-124	1.00E-01	3.09E-109
H4k20me1	9.83E-02	1.02E-159	9.84E-02	1.17E-156
Ctcf	1.00E-01	8.67E-40	9.97E-02	2.07E-69
H3k27ac	1.00E-01	2.10E-80	1.00E-01	3.51E-104
H3k27me3	***1.00E-01***	***2.71E-01***	1.00E-01	9.59E-94
H3k36me3	1.00E-01	3.79E-88	9.98E-02	1.32E-126
H3k4me1	1.00E-01	1.01E-52	1.00E-01	3.60E-112
H3k4me2	**1.01E-01**	**1.64E-14**	9.97E-02	3.31E-135
H3k4me3	1.00E-01	7.32E-04	1.00E-01	5.05E-86
H3k9ac	1.00E-01	8.62E-48	1.00E-01	1.08E-102
